# Twisting the TAPPs: *Bay*‐Substituted Non‐planar Tetraazapero‐pyrenes and their Reduced Anions

**DOI:** 10.1002/chem.201903413

**Published:** 2019-10-22

**Authors:** Benjamin A. R. Günther, Sebastian Höfener, Ute Zschieschang, Hubert Wadepohl, Hagen Klauk, Lutz H. Gade

**Affiliations:** ^1^ Anorganisch-Chemisches-Institut Universität Heidelberg Im Neuenheimer Feld 270 69120 Heidelberg Germany; ^2^ Institute of Physical Chemistry Karlsruhe Institute of Technology (KIT) P.O. Box 6980 76049 Karlsruhe Germany; ^3^ Max Planck Institute for Solid State Research Heisenbergstr. 1 70569 Stuttgart Germany

**Keywords:** halogens, perylenes, radicals, synthetic methods, UV/Vis spectroscopy

## Abstract

A new synthesis of tetraazaperopyrenes (TAPPs) starting from a halogenated perylene derivative 3,4,9,10‐ tetrabromo‐1,6,7,12‐tetrachloroperylene (**1**) gave access to *bay*‐substituted TAPPs for the first time. Selective lithiation of the bromine‐positions and subsequent addition of tosyl azide led to the formation of the tetraazidotetrachloroperylene (**2**), which was subsequently reduced by addition of sodium borohydride to the corresponding tetraaminotetrachloroperylene (**3**). Oxidation to its semiquinoidal form **4** and subsequent cyclization with acid chlorides gave rise to a series of *bay*‐chlorinated TAPPs. Whereas the aromatic core of the previously studied *ortho*‐substituted TAPPs was found to be planar, the steric pressure of the two chlorine substituents on each side leads to the twist of the peropyrene core of approximately 30 degrees, a structural feature also observed in other *bay*‐substituted perylene derivatives. An experimental and computational analysis reveals that introducing chloride substituents at these positions leads to slightly increased electron affinities (EA) enabling the selective generation and characterization of the reduced mono‐anionic radicals and closed shell di‐anionic species. These anions were isolated and characterized by UV/Vis spectroscopy and EPR or NMR, respectively. Processing of the *bay*‐chlorinated TAPPs in n‐channel organic TFTs revealed electron mobilities of 0.001 to 0.003 cm^2^ V^−1^ s^−1^. These reduced electron mobilities compared to the *ortho*‐halogenated TAPPs are thought to be rooted in the less densely packed solid‐state structures.

## Graphical abstract

**First twisted—then TAPPed**: Previously inaccessible *bay*‐substituted tetratazaperopyrenes (TAPPs) were obtained from “twisted” perylene‐containing *bay*‐substituents. The resulting TAPPs were found to be readily reduced to their radical monoanions and closed shell dianions.

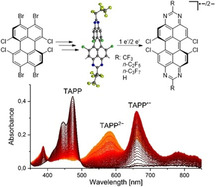

## Introduction

Perylene tetracarboxydiimides (PDIs) have been extensively studied as functional dyes and electronic materials.[^1^, ^2^, ^3^, ^4^, ^5^, ^6^, ^7^, ^8^, ^9^, ^10^, ^11^, ^12^, ^13^, ^14^, ^15^, ^16^, ^17^] Their properties may be varied widely by substitution at the perylene core and at the imido‐N position. The carboxy substituents at the central perylene core render these materials electron acceptors, and this property in particular has been underlying their application in organic electronics.

**Figure 1:**
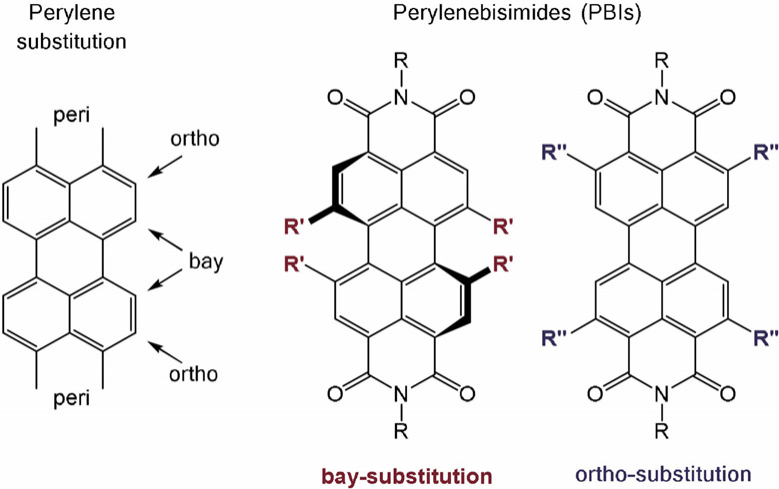
Left: Substituent positions in perylene derivatives. Right: *bay*‐ and *ortho* substitution in PDIs.

Different synthetic approaches have been established to functionalize the *bay*‐[^2^, ^10^, ^18^, ^19^, ^20^, ^21^, ^22^, ^23^] or *ortho*‐[^24^, ^25^, ^26^, ^27^, ^28^, ^29^, ^30^, ^31^] positions selectively or even to fully substitute[^26^, ^32^, ^33^] the perylene core in a single‐step‐reaction (Figure 1).[^34^, ^35^]

A class of molecular dyes which has displayed photophysical and redox properties similar to the ubiquitous PDIs are the tetraazaperopyrenes (TAPPs) which have been investigated for more than a decade.[^36^, ^37^, ^38^, ^39^, ^40^, ^41^, ^42^, ^43^, ^44^, ^45^, ^46^, ^47^, ^48^] Whereas the electron accepting character of the PDIs is due to carboximide substituents at both ends of the perylene core, TAPPs contain two pyrimidine rings fused with the central perylene unit. The electron accepting character of the resulting N‐heteropolycycles is thus effectively built into the aromatic core itself rather than substituent‐induced. Viewed alternatively, the fourfold isosteric [CH→N] substitution in the parent hydrocarbon peropyrene radically changes its electronic properties while leaving its molecular shape practically unchanged.

**Scheme 1:**
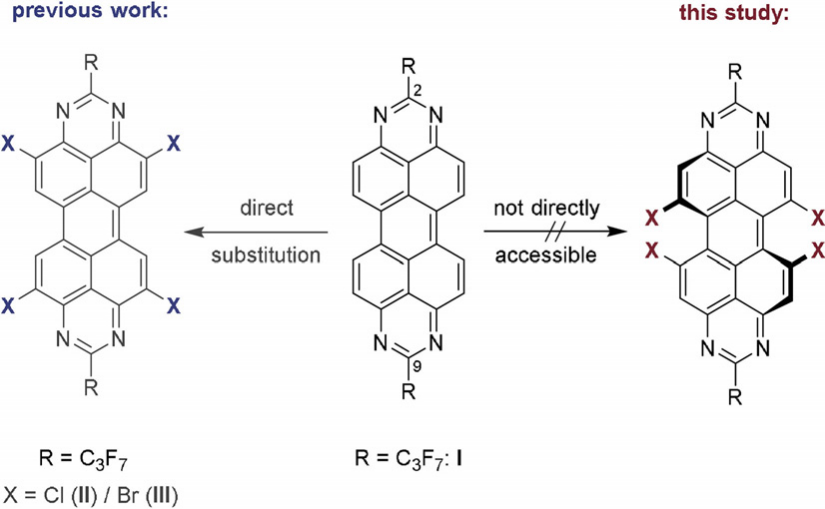
Approach to synthesize *ortho*‐substituted TAPP‐derivatives (left, previous work) and inability to functionalize the *bay*‐position (right) when starting from earlier parent compound **I**.

Whereas electrophilic substitution of PDIs and related compounds occurs in the *bay*‐positions, the corresponding derivatization of the parent TAPP compounds (**I**) happened exclusively in the *ortho*‐position, leaving the *bay*‐CH units unaffected and thus maintaining the planar structure of the aromatic core (Scheme 1).[^42^, ^44^] Manifold derivatization of TAPP derivatives in the 2,9‐positions, as well as in the *ortho*‐position, has led to a detailed understanding of the molecular behavior and its photophysical and electronic properties. On the other hand, it has not yet been possible to functionalize the inner *bay* position of the peropyrene core, in order to establish a link with the well established PDI chemistry and its further development towards more complex polycyclic aromatics. Thus, a fundamental part of TAPP‐chemistry has remained unexplored to date.

In this work, we report a novel TAPP synthesis, which for the first time allowed their *bay* functionalization. Additionally, a detailed study of their reduced species was conducted to gain insights into electronic behavior and their stability. Comparison of the *ortho*‐ and *bay*‐chlorinated TAPPs provided insight into the influence of the different substitution patterns of the peropyrene core.

## Results and Discussion

### Synthesis of *bay*‐chlorinated TAPPs

**Scheme 2:**
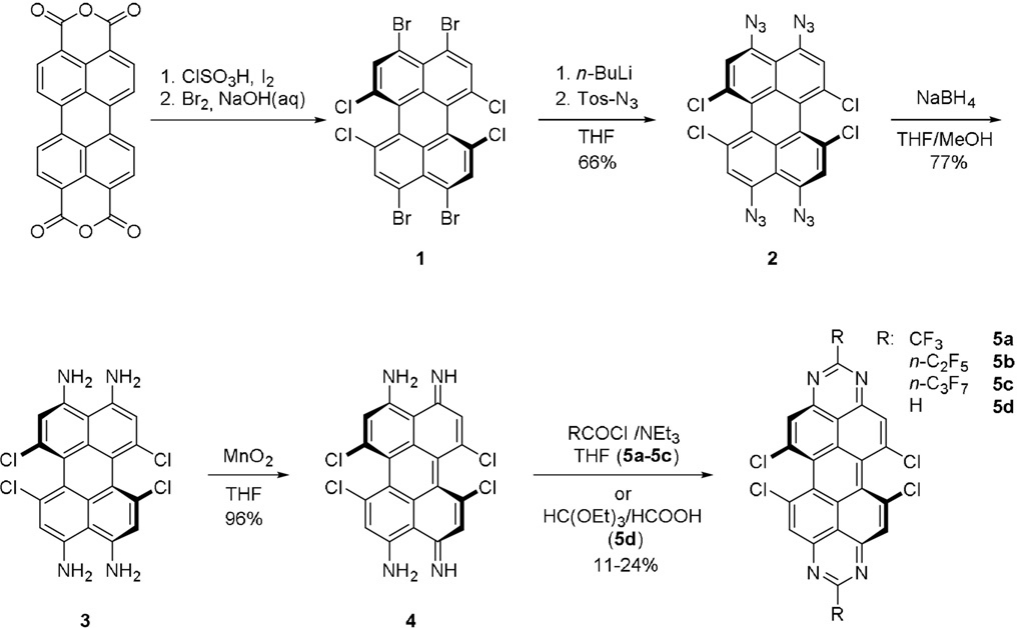
Synthetic route to the *bay*‐chlorinated DPDI analogue (**DPDI‐Cl**, **4**) and subsequent ring‐closing reaction to the corresponding *bay*‐TAPP‐Cl **5**.

The starting material for the synthesis of *bay*‐substituted TAPPs was tetrabromotetrachloroperylene (**1**), first published in 2014 (Scheme 2).^[49]^ Selective lithiation of the bromine positions and subsequent addition of tosyl azide led to the formation of the tetraazidotetrachloroperylene (**2**), which was subsequently reduced by addition of sodium borohydride to the corresponding tetraaminotetrachloroperylene (**TAP‐Cl**, **3**). Analogous to the previously reported non‐chlorinated tetraaminoperylene, this compound displayed a tendency to be oxidized under ambient conditions to form the chlorinated diaminoperylenequinone‐diimine (**DPDI‐Cl**, **4**), however, the reaction proceeded much more slowly than for the parent **DPDI** and occasionally did not go to completion. Therefore, oxidation to the semiquinoidal compound **4** was selectively carried out by the reaction of **3** with activated MnO_2_.

By employing the standard ring‐closing reaction conditions of the original TAPP‐synthesis, the first *bay*‐substituted TAPP derivatives with H‐ or perfluoroalkyl‐substituted 2‐ and 9‐position (**5**) were obtained. As opposed to the planar *ortho*‐substituted TAPP derivatives, which only possess low solubility in common organic solvents, the twist induced by the *bay*‐chlorination leads to a significantly enhanced solubility.

### Crystal structures of 5 a–c

**Figure 2:**
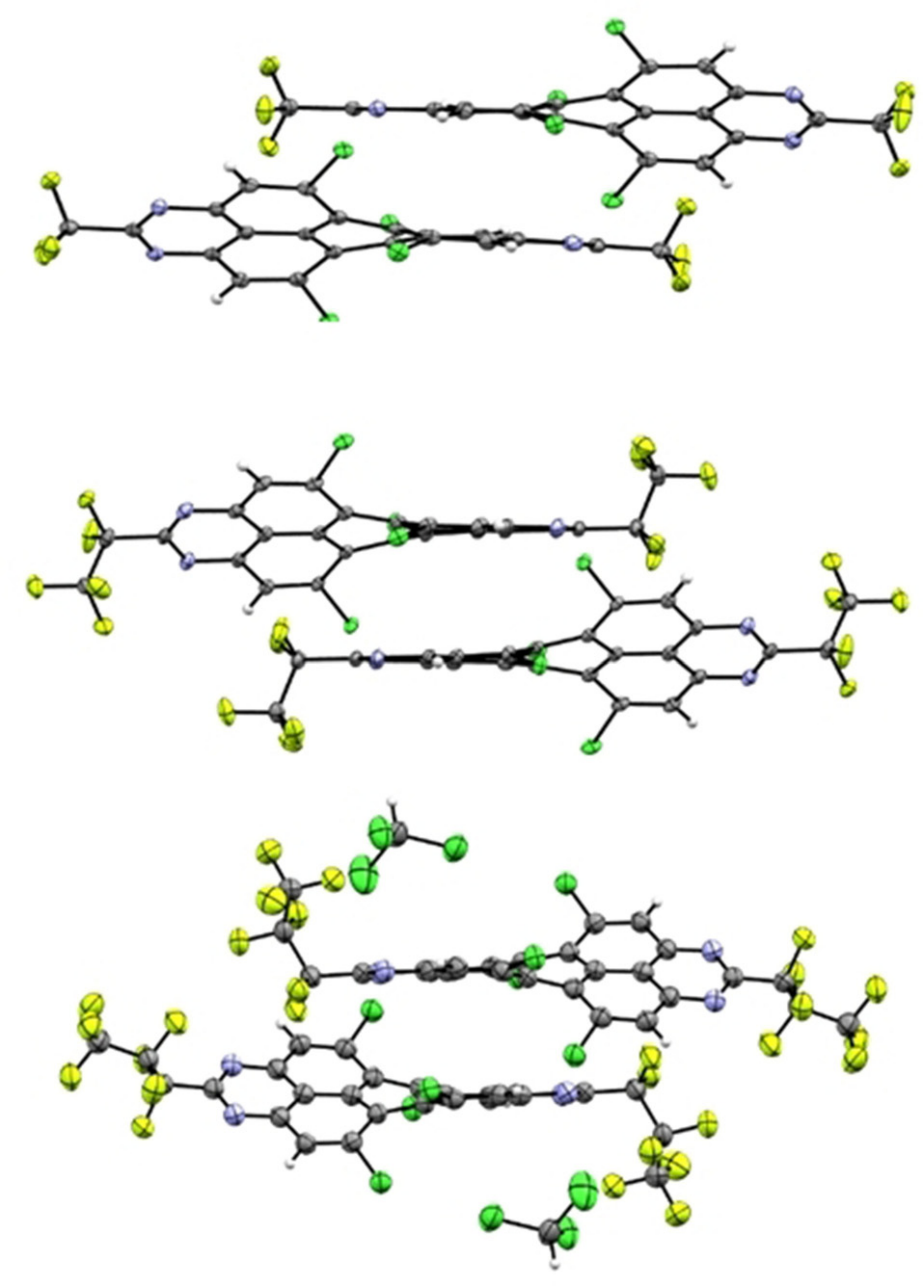
Solid state structure of **5 a** (top), **5 b** (middle) and **5 c** (bottom).

Single crystals of the tetrachlorinated TAPPs **5 a**, **5 b** and **5 c** were grown by slow evaporation of concentrated solutions in chloroform (**5 a** and **5 c**) and toluene (**5 b**) (Figure 2). All three solid‐state structures reveal a significant twist between the two diazaphenalenyl subunits of the tetraazaperopyrene core: **5 a** (29.4 degrees), **5 b** (30.6 degrees) and **5 c** (32.4 degrees). Whereas the aromatic core of the previously studied *ortho*‐substituted TAPPs was found to be planar, the steric pressure of the two chlorine substituents on each side leads to the twist of the peropyrene core, a structural feature also observed in other *bay*‐substituted perylene derivatives.^[18]^

**Figure 3:**
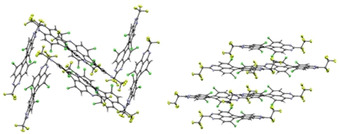
Packing pattern of **5 a** in a zigzag‐like fashion (left) and of **5 b** in a columnar pattern (right).

In all three cases, the packing pattern in the solid state is determined by intermolecular stacking of two diazaphenalenyl subunits. This motif leads to distances as close as 3.4 Å between the aromatic core halves of two adjacent molecules, however, the general packing pattern varies for the different perfluorinated alkyl chains (Figure 3). Thus, the CF_3_‐substituted TAPP **5 a** packs in a zigzag fashion, while the elongation of the fluoroalkyl chains in **5 b** leads to more columnar‐like packing pattern. The crystals of **5 c** could not be obtained without co‐crystallization of solvent, which renders an assessment of the packing pattern less significant. The key data of the solid‐state structures are summarized in Table 1.

**Table 1:** Structural parameters of solid‐state structures of **5 a**–**c**.

	Crystal system	Space group	π–π Plane distance [Å]^[a]^	Torsion angle [°]^[b]^
**5 a**	orthorhombic	*Pbca*	3.440	29.43 (0.03)
**5 b**	monoclinic	*I*2/*a*	3.535	30.10 (0.03)
**5 c**	triclinic	*P* $\bar{1}$	3.603^[c]^	32.44 (0.07)

[a] π–π Distance was measured between two adjacent diazaphenalenyl‐subunits. [b] Torsion angle was measured between both diazaphenalenyl‐subunits of one molecule. [c] **5 c** co‐crystallized with chloroform, but displaying close packing between pairs of molecules.

### Interconversion barrier of atropo‐enantiomers

**Scheme 3:**
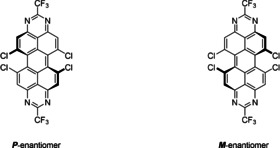
The two possible *P*‐and *M*‐enantiomers of *bay*‐substituted TAPPs.

As the steric repulsion between opposing chlorine atoms leads to the aforementioned torsion in the peropyrene backbone, the dye molecules possess helical chirality, leading to *P*‐ and *M*‐atropoisomers (Scheme 3). This helical isomerism has been studied in detail for the structurally similar *bay*‐functionalized perylene bisimides,[^23^, ^50^, ^51^, ^52^, ^53^, ^54^, ^55^, ^56^, ^57^, ^58^] and the interconversion barrier between the atropo‐enantiomers was found to depend on the number and steric demand of the *bay* substituents. Whereas for most PDIs interconversion occurs rapidly at room temperature, larger substituents raise the energy barrier over 22.2 kcal mol^−1^, which is the minimum barrier required for the isolation of the enantiomers at ambient temperature and the usual timescale of product handling. It was therefore of interest to compare analogously substituted PDI‐ and TAPP‐systems in order to obtain insight into the flexibility of the peropyrene core compared to the PDI system.[^54^, ^59^]

**Scheme 4:**
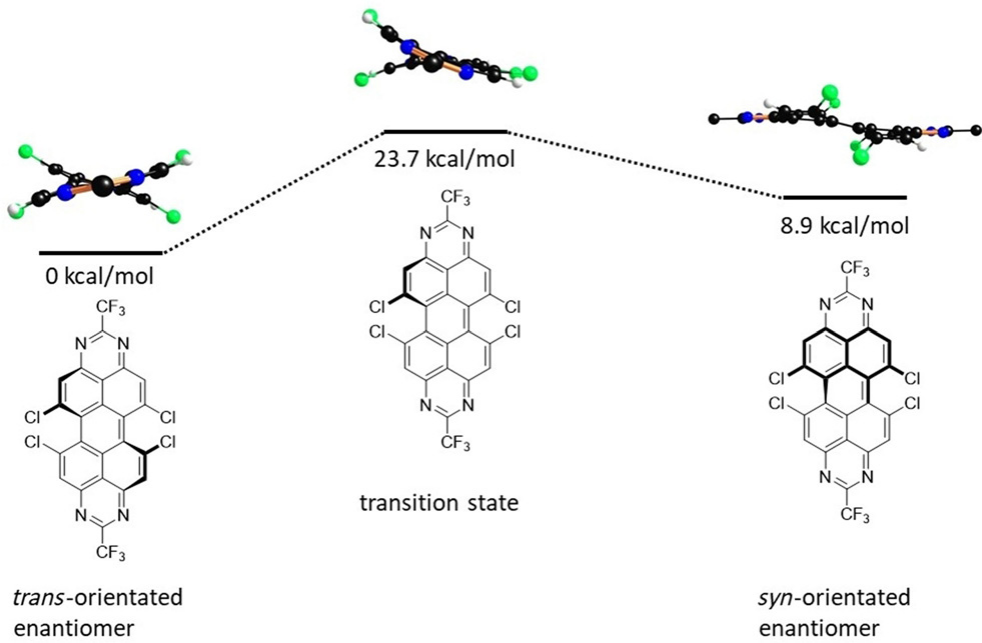
Calculated relative energies of conversion between atropo‐isomers in kcal mol^−1^.

To this end, the interconversion of the atropisomeric *bay*‐chlorinated tetraazaperopyrenes, has been modeled theoretically by DFT (Scheme 4). For a potential one‐step mechanism involving a planar transition state the energy barrier would be approximately 72 kcal mol^−1^, thus locking the molecules in a single helical form. As an alternative pathway, the stepwise isomerization was investigated, in which the first transition state is reached via the planarization on only one side of the TAPP, while the opposite side remains in the twisted form. This results in a reduced torsion angle of about 10 degrees compared to 32 degrees in the ground state (Table 2). The computed activation energy was found to be 23.7 kcal mol^−1^, and the resulting intermediate *bay*‐substituted TAPP is characterized by mutually *syn*‐oriented chloro substituents. This achiral intermediate is only 9 kcal mol^−1^ higher in energy than the *trans*‐oriented conformer and either reacts back to the initial form or to the other atropisomer.

**Table 2:** Computed relative energies including the zero‐point vibrational energy (ZPVE) and angles of intermediates and the transition state (TS) structure.

	*E*_ZPVE_ [kcal mol^−1^]	Torsion angle [°]^[a]^	Imaginary frequency [cm^−1^]
***trans*** **‐oriented enantiomer**	0	32	
**TS**	24	10/31	74.96
***syn*** **‐oriented intermediate**	9	23

[a] Torsion angle was measured using the four C‐atoms in the *bay*‐area.

This reaction sequence corresponds to the mechanism, Würthner et al. have proposed for the corresponding PDI‐systems.^[54]^ Notably, the isomerization barrier of the *bay*‐chlorinated TAPPs is very similar to that reported for the PDI‐Cl_4_, which suggests that indeed the PDI‐core possesses similar rigidity/flexibilty as the polycyclic aromatic core of the TAPP molecules.

### UV/Vis absorption and emission spectra

**Figure 4:**
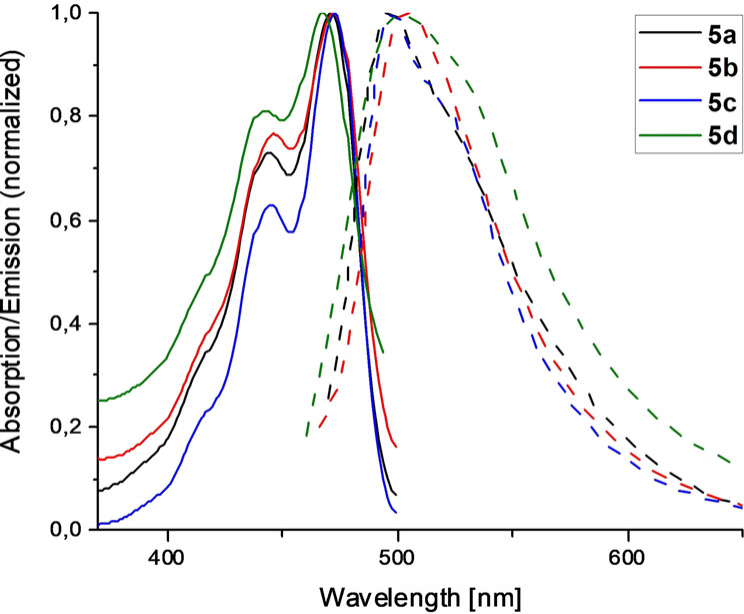
Absorption (continuous line) and emission (dashed line) spectra of *bay*‐substituted compounds **5 a**–**d** recorded in THF.

The UV/Vis absorption and emission spectra of the TAPP derivatives **5 a**–**d** recorded in THF are displayed in Figure 4. Similar to the non‐twisted *ortho*‐substituted TAPP derivatives, the absorption and emission maxima remain unchanged upon variation of the alkyl‐substituents in the 2‐ and 9‐positions.^[42]^

All derivatives were found to exhibit absorption maxima in the range of 468 to 474 nm with vibrational progressions and emission maxima in the range of 496 to 505 nm. As TAPPs are known to aggregate in solution, measurements were repeated in different solvents (DCM and CHCl_3_) and in different concentrations. However, identical absorption and emission data were observed, leading to the conclusion, that these twisted TAPPs do not tend aggregate in common organic solvents. An overview of the optical properties is given in Table 3.

**Table 3:** Photophysical properties of **5 a**–**d** measured in THF and computed vertical electronic excitation (VE) and de‐excitation (VD) energies.

	R	*λ*_abs_ [nm] (log *ϵ*)^[a]^	*λ*_em_ [nm] (*ϕ*_Em_)^[a]^	0–0 Transition [nm]^[b]^	Stokes shift [cm^−1^]	*λ*_VE_ [nm] (DFT)^[c]^	*λ*_VD_ [nm] (DFT)^[c]^
**5 a**	CF_3_	471 (4.57)	496 (0.12)	482	1070	481	534
**5 b**	C_2_F_5_	472 (4.64)	505 (0.09)	485	1384	482	535
**5 c**	C_3_F_7_	474 (4.59)	496 (0.10)	484	936	482	536
**5 d**	H	468 (3.98)	501 (0.05)	480	1407	478	532

[a] Absorption and emission spectra were recorded in THF. [b] 0‐0‐Transition was obtained of the intersection of absorption and emission spectra. [c] Vertical electronic excitation (VE) and de‐excitation (VD) energies were calculated using B3LYP as functional and TZVPP as the basis set.

Complementing the measurement of the absorption and emission spectra in solution, Table 3 contains vertical transition energies computed at time‐dependent density functional theory (TDDFT) level of theory. While the absolute values of the vertical excitation (VE) and the vertical de‐excitation (VD) energies deviate from the measured absorption and emission bands by 0.05 and 0.15 eV, respectively, the table confirms that the photophysical data remain unaffected by the variation of substituents in the 2‐ and 9‐positions.

**Figure 5:**
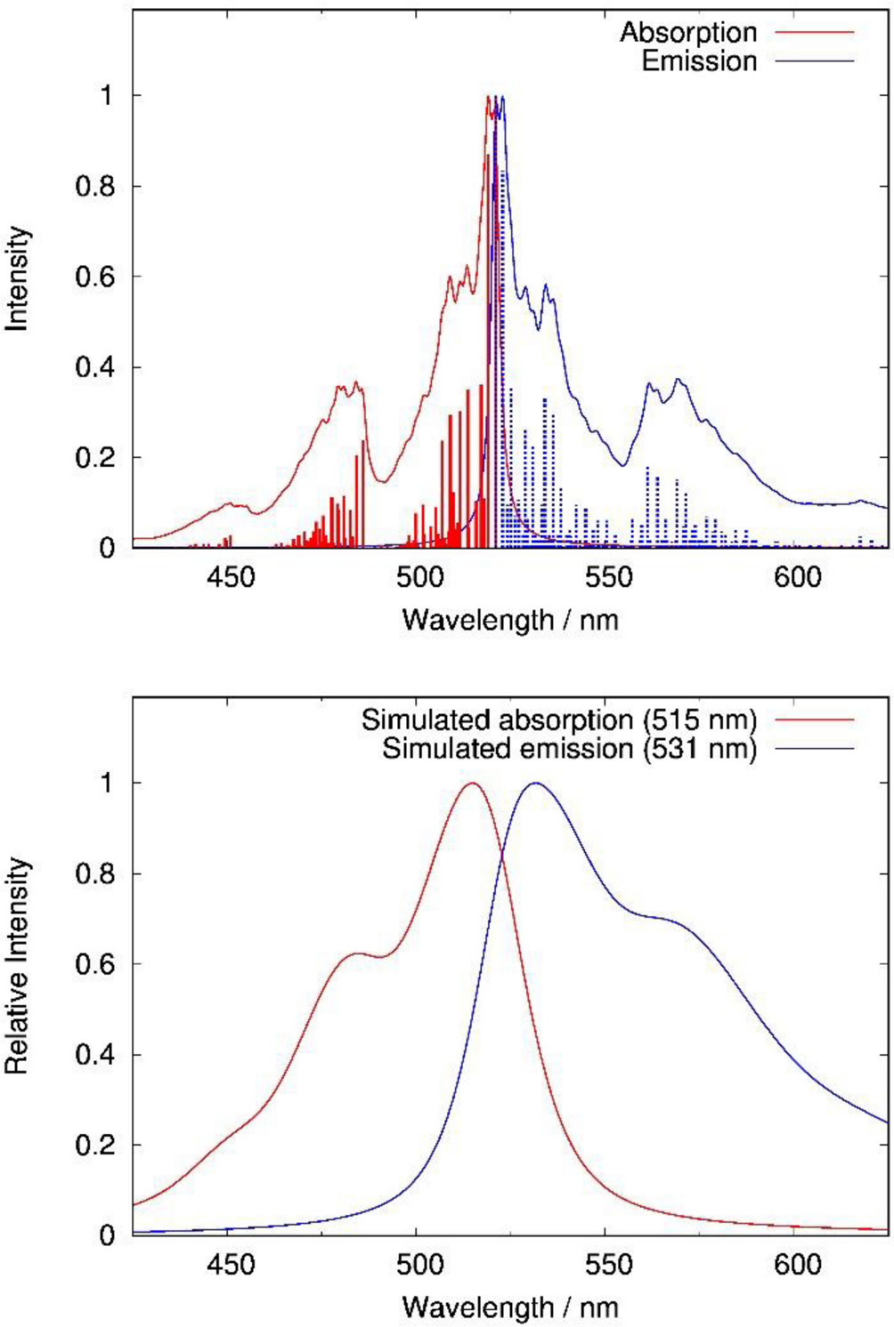
Simulated spectra of compound **5 d** at 0 K (top) and 300 K (bottom), shifted according to the theoretical 0‐0 transition energy of 521 nm (2.38 eV), computed in the harmonic approximation at B3LYP/def2‐TZVPP level of theory employing *D*_2_ symmetry.

Since computed vertical electronic transition energies cannot be directly related to the experimental results, we also simulated vibrationally resolved adiabatic transitions.^[48]^ Simulated absorption and emission spectra of **5 d** are displayed in Figure 5. At zero temperature, the simulated spectra show distinct transitions for individual vibronic transitions.

The spectra do not seem to be dominated by the 0–0 transition and they thus display a Stokes shift of 0.02 eV (140 cm^−1^) at zero temperature, however, the incorporation of temperature effects leads to a Stokes shift of about 0.07 eV (about 586 cm^−1^) at 300 K. The finite‐temperature spectrum furthermore reproduces the general shape of the experimental spectra as shown in Figure 4, which is characterized by a pronounced vibrational progression at lower wavelength for the absorption but only a broadened band for emission. We note that the experimentally observed Stokes shifts are larger compared to the simulated values, which might be due to solvation effects which are not included in the simulations in vacuum.

### Redox properties

**Figure 6:**
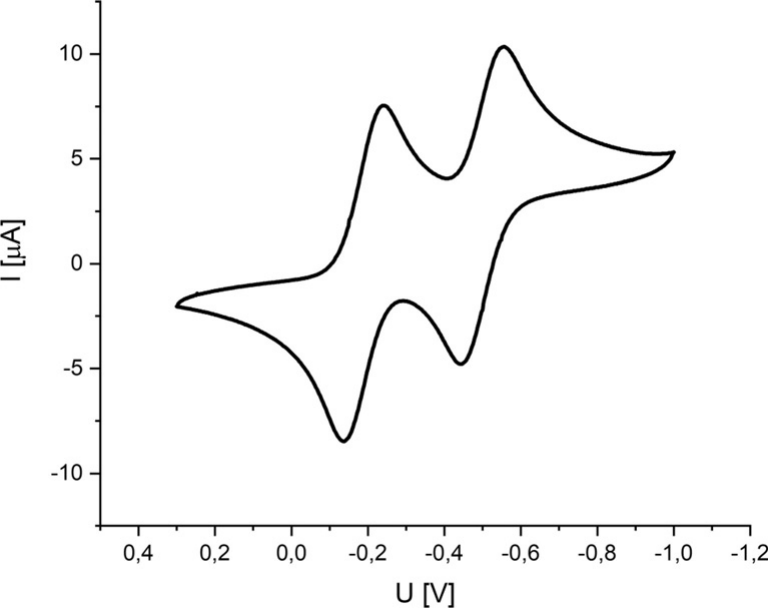
Cyclic Voltammogram of **5 b**, measured in DCM, supp. electrolyte Bu_4_NPF_6_, reference SCE, sweep rate 50 mV s^−1^.

To assess the redox chemical properties of the *bay*‐chlorinated TAPP derivatives cyclic voltammograms (CVs) of all compounds were recorded. They were found to exhibit two individual and fully reversible one‐electron reduction steps to the mono‐anionic radical species and the subsequent di‐anionic compound. In Figure 6, the CV is shown for compound **5 b**.

The energies of the lowest unoccupied molecular orbital (LUMO) were estimated by subtraction of the reversible first reduction potentials obtained from the cyclic voltammograms of the redox potential of ferrocene (4.8 eV for Fc/FC^+^).^[60]^ As expected, the substituents in the 2‐ and 9‐position have only limited influence on the frontier orbitals, similar to the UV/Vis absorption and emission spectra discussed above. This was backed up by the computational determination of the Kohn–Sham frontier orbital energies determined by DFT.

**Figure 7:**
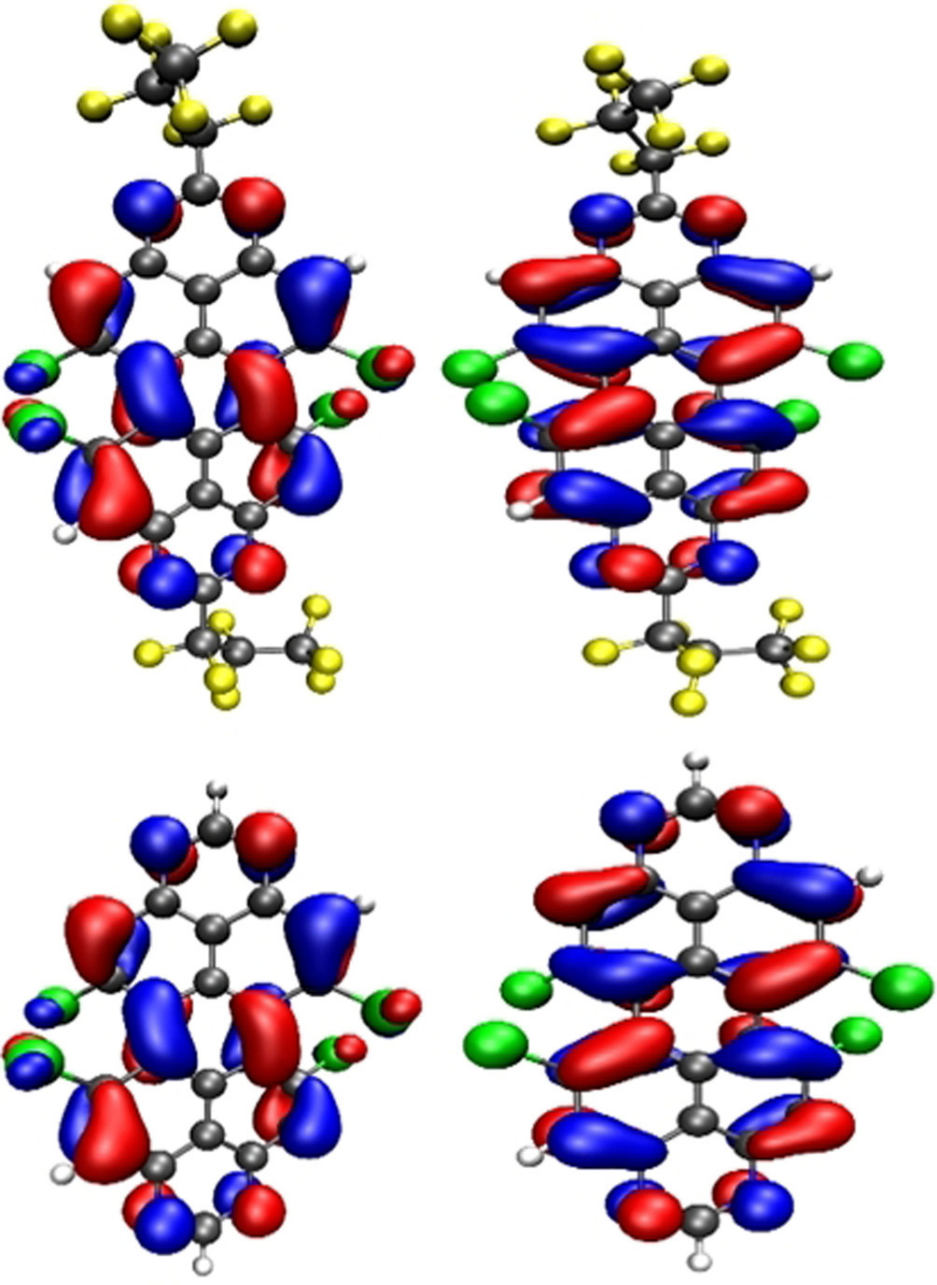
Visualization of the HOMO (left) and LUMO (right) of **5 c** (top) and **5 d** (bottom); level of theory used is B3LYP/def2‐SVP, isovalue 0.03.

Whereas the energies of the LUMO and the highest occupied molecular orbital (HOMO) of derivatives **5 a**–**c** are almost identical at around −4.15 eV, the “removal” of the perfluorinated alkyl group leads to an increase of both the HOMO and LUMO energies by about 0.5 eV, resulting in a virtual identical HOMO–LUMO gap of all derivatives. Visualization of the frontier orbitals (Figure 7), reveals the nodal planes of the frontier orbitals along the principal molecular axis in which the substituents in the 2‐ and 9‐positions are located. This also explains the nearly identical absorption and emission spectra of **5 a**–**d** which are dominated by the HOMO–LUMO transition.

For the perfluorinated alkyl groups electron affinities between 3.21 and 3.31 eV were found for **5 a**–**c**, whereas the hydrogen‐substituted compound **5 d** was found to possess a significantly reduced electron affinity of about 2.73 eV (Table 4). These adiabatic EA values do not differ significantly from vertical EA values obtained from GW methods (The GW method is a way to correct artefacts in Kohn–Sham (KS) methods which employ exchange‐correlation (XC) functionals),^[61]^ see Table 2, which indicates that the one‐electron reduction does not induce a significant change in the molecular geometry. This corresponds to a small reorganization energy which is desirable for electron transporting compounds with large *λ*_+_ (reorganization energy for hole transport) and small *λ*_−_(reorganization energy for electron transport)_._ ^[62]^

**Table 4:** Adiabatic electron affinities (aEA) obtained from the Δ*S*CF approach, vertical electron affinities (vEA) from the GW method, and frontier orbital energies of compounds **5 a**–**d**.

	R	aEA _(Δ*S*CF)_ [eV]^[a]^	vEA _(GW)_ [eV]^[a]^	*E*_HOMO (DFT)_ [eV]^[a]^	*E*_HOMO–LUMO_ [eV]	*E*_LUMO (DFT)_ [eV]^[a]^	*E*_LUMO (exp, CV)_ [eV]^]b]^
**5 a**	CF_3_	3.23	3.21	−6.84	2.71	−4.12	−4.14
**5 b**	C_2_F_5_	3.22	3.25	−6.86	2.71	−4.15	−4.21
**5 c**	C_3_F_7_	3.31	3.27	−6.91	2.71	−4.20	−4.20
**5 d**	H	2.73	2.75	−6.43	2.72	−3.71	−3.93

[a] Properties were calculated using B3LYP as the functional and a def2‐QZVPP as basis set. [b] LUMO energy was estimated from the reversible first reduction potentials obtained from the cyclic voltammograms using the redox potential of ferrocene as reference (4.8 eV for Fc/Fc^+^).^[60]^

**Figure 8:**
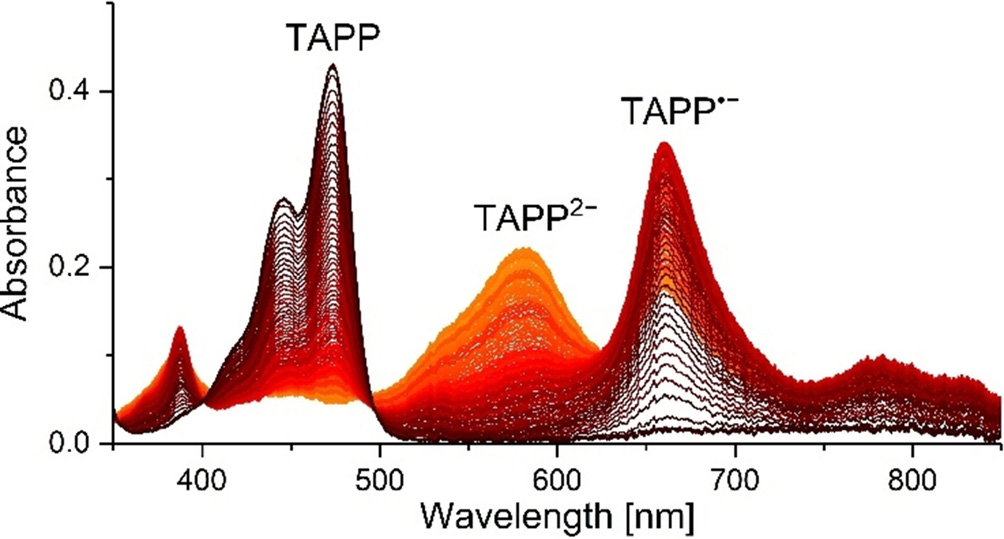
Absorption spectra of the two individual reduction steps of **5 a** in THF; voltage steps from 0 V (black) via −0.5 V (red) to −1.0 V (orange).

The two individual one‐electron reduction steps occurring consecutively were monitored for **5 a** by UV/Vis spectroscopy in a spectroelectrochemical cell (Figure 8). By applying a potential of −0.5 V the absorption band at 474 nm of the neutral compound completely disappeared, while the formation of a green species with a long‐wavelength absorption maximum at 663 nm was detected. This can be attributed to the monoanionic radical TAPP^.−^. The further lowering of the reduction potential to −1.0 V led to the second reduction step, observable by a change of color to purple and the hypsochromic shift of the absorption band to 663 nm. The spectra exhibit relatively well defined isosbestic points at 400 nm and 495 nm, indicating that both reduction steps proceed cleanly and without significant further conversions.

**Scheme 5:**
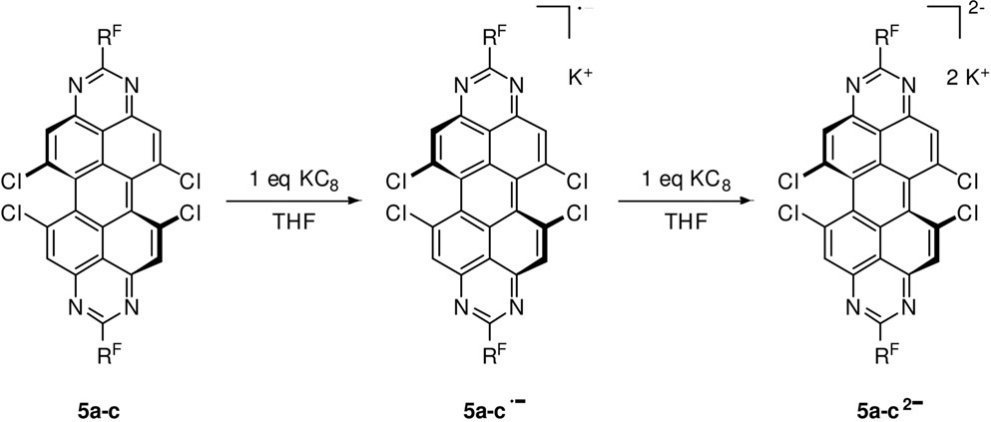
Reduction of **5 a**–**c** to the radical monoanionic (**5 a**—**c^.−^)** or diamagnetic dianionic TAPP (**5 a**–**c^2−^**) using KC_8_.

**Figure 9:**
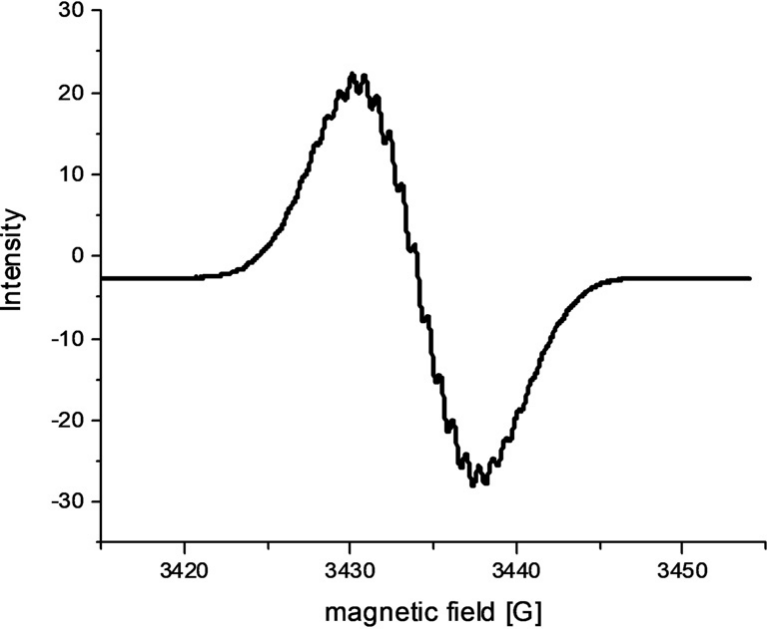
EPR‐spectrum (THF, r.t.) of **5 b**.

The two reduced TAPP species, TAPP^.−^ and TAPP^2−^, of **5 a**–**5 c** could also be isolated quantitatively by addition of one or two equivalents of KC_8_, respectively (Scheme 5). The radical monoanionic TAPP‐compounds were characterized by EPR (Figure 9), which for all derivatives possess a *g*‐factor of 2.004, which is to be expected for radical organic compounds. Unfortunately, hyperfine couplings were only partially resolved precluding a complete simulation of the spectra.

None of the radical monoanions showed any signs of degradation under inert atmosphere, and even under ambient conditions the radicals only degraded very slowly, over a period of weeks, in the solid state. The insertion of the second electron led to a diamagnetic species, which allowed the compounds to be characterized by standard NMR methods, performed exemplarily for compound **5 a**. Both ^1^H NMR spectra and ^19^F NMR spectra display a shift of their signals to a higher field probably due to the enhanced electronic shielding by the additional electrons (Supporting Information). Also, the UV/Vis absorption spectra were recorded for each species, which reproduced the results of the spectroelectrochemical investigation depicted in Figure 8 (Supporting Information).

### *Bay*‐chlorinated TAPPs as n‐channel semiconductors

Ortho‐substituted TAPP derivatives have been successfully employed as semiconductors in n‐channel organic TFTs, exhibiting electron mobilities of up to 0.17 cm^2^ V^−1^ s^−1^.^[63]^ In analogy, the *bay*‐substituted TAPPs **5 a**–**c** were evaluated in bottom‐gate, top‐contact TFTs with a vacuum‐deposited semiconductor layer (Table 5). The TFTs consisted of a doped silicon substrate that also served as a common gate electrode, a stack of thermally grown SiO_2_, atomic‐layer‐deposited Al_2_O_3_ and a self‐assembled monolayer (SAM) of *n*‐tetradecylphosphonic acid as the gate dielectric, and shadow‐mask‐patterned Au top contacts (a detailed description of the fabrication process can be found in the Supporting Information). In all cases, n‐channel semiconducting behavior was observed, with electron mobilities of 0.001 to 0.003 cm^2^ V^−1^ s^−1^.

**Table 5:** Summary of the transistor parameters electron field‐effect mobility (*μ_n_*), on/off current ratio (*I*_on_/*I*_off_), threshold voltage (*V*_th_), subthreshold swing (*SS*) and temperature (*T*) measured under ambient conditions.

	*I*_on_/*I*_off_	*V*_th_[V]	*SS* [V dec^−1^]	*T* [°C]	*μ*_n_ [cm^2^ V s^−1^]
**5 a**	10^4^	6	1.9	70	0.002
**5 b**	10^4^	19	2.8	40	0.003
**5 c**	10^4^	13	4.4	90	0.001

**Figure 10:**
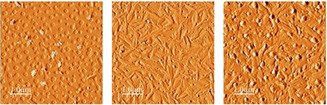
Atomic force microscopy (AFM) images of vacuum‐deposited films of **5 a** (left), **5 b** (middle) and **5 c** (right).

The atomic force microscopy (AFM) images of the vacuum‐deposited TAPP films reveal a low tendency of self‐organization. The films of **5 a** and **5 c** are essentially amorphous, and only **5 b** shows a locally crystalline structure in certain areas of the image (Figure 10), which may explain the relatively low electron mobilities.

In all cases, variation of the substrate temperature during the semiconductor deposition did not lead to an improvement of the self‐organization of the material. On the other hand, the reduced tendency to pack in tight units, however, may open opportunities for the TAPP chemistry to explore new fields of applications. Also, the increased solubility associated with the reduced packing density is expected to be beneficial for solution processing.

### *Ortho*‐ versus *bay*‐substitution

**Figure 11:**
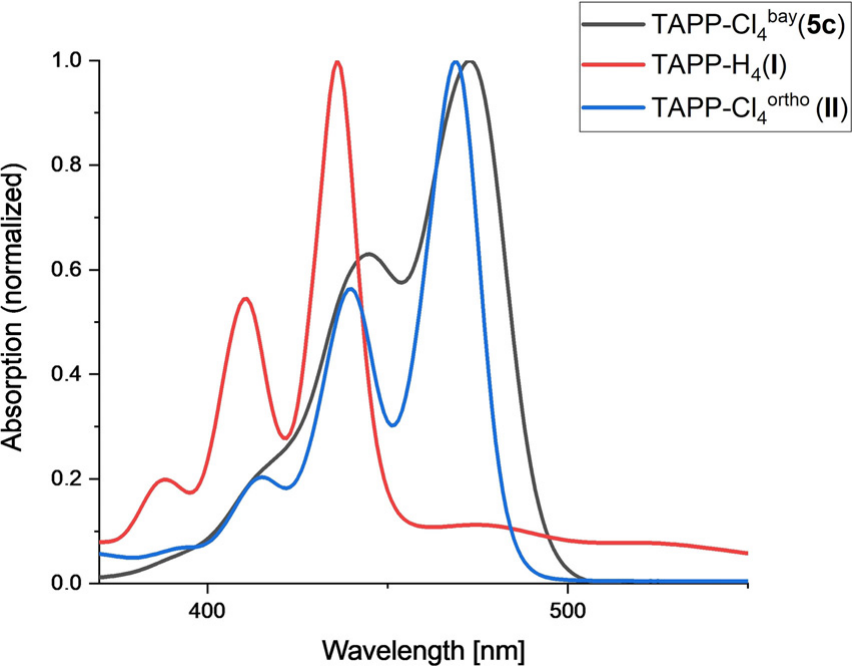
Absorption spectra of unsubstituted TAPP **I** (red), *ortho*‐chlorinated TAPP **II** (blue) and *bay*‐chlorinated TAPP **5 c** (black) in THF.

To further understand the different influence of *ortho*‐ and *bay*‐substitution in TAPP derivatives, the two core‐chlorinated TAPP isomers **5 c** and **II** shall be compared. The absorption maxima at 474 nm (**5 c**) and 469 nm (**II**) of both isomers indicate that the bathochromic shift, induced by the chlorine substitution, is almost equally pronounced (Figure 11). However, the emission maximum of the *bay*‐substituted derivative is red‐shifted by 15 nm compared to the *ortho*‐substituted TAPP, resulting in a Stokes shift for **5 c** of 936 cm^−1^ which is almost twice as large as observed for **II** (445 cm^−1^). Additionally, the high fluorescence quantum yield of the *ortho*‐chlorinated **II** (0.78) is reduced to 0.10 for the twisted *bay*‐chlorinated TAPP **5 c** which appears to be characterized by more accessible internal degrees of freedom and thus enhanced non‐radiative relaxation processes.

**Figure 12:**
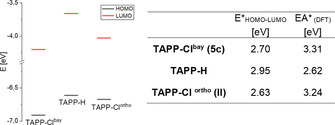
Calculated HOMO‐ and LUMO‐levels (left) and HOMO–LUMO‐gaps and EA (right) of **I**, **II** and **5 c**.

The comparison of the electrochemical properties also reveals some significant differences between **5 c** and **II** (Figure 12). The LUMO‐level of both chlorinated isomers at −4.20 eV (**5 c**) and −4.03 eV (**II**) is significantly stabilized compared to the unsubstituted compound **I** (−3.66 eV), however, this effect is even more pronounced for the *bay*‐substituted TAPP **5 c**. In contrast to the *ortho*‐chlorinated derivative **II**, the *bay*‐substitution additionally leads to a stabilization of the HOMO level, leading again to similar HOMO–LUMO gaps of 2.70 eV (**5 c**) and 2.63 eV (**II**). The electron affinity, which is also a key parameter in the evaluation of potential n‐channel semiconductors, is higher for the *bay* substitution (3.31 eV) compared to the *ortho* substitution (3.09 eV). Overall, *bay*‐chlorination leads to a greater stability of the anionic (radical) species compared to the *ortho*‐substituted constitutional isomers.

Whereas both, the photophysical and the electronic properties are similar for both TAPP‐Cl isomers, the change of molecular geometry resulting from the different substitution patterns, is significant. Whereas the planar peropyrene core is barely affected by the *ortho*‐substitution, the steric repulsion of the *bay* substituents leads to a large twist of approximately 30 degrees between the two subunits of the TAPP. This difference in molecular structure gives rise to an organized and dense packing pattern in case of the *ortho*‐substituted derivative **II** in its solid‐state structure (with intermolecular distances of 3.38 Å), whereas this type of packing is disrupted for the *bay*‐substituted derivative **5 c** leading to reduced electron mobilities while the molecular redox properties are virtually unchanged. Conversely, the reduced long‐range order, leads to a significantly increased solubility and potential processability of the *bay*‐substituted molecules.

## Conclusions

In this study we have expanded the chemistry of tetraazaperopyrenes (TAPPs) to include non‐planar *bay*‐chlorinated derivatives. This required a new synthetic strategy which was based on previous work in perylene chemistry and required the introduction of the condensed pyrimidine units to the central perylene core in the final reaction steps. As expected from previous studies of perylene bisimides, the *bay*‐chlorinated TAPPs possess a twisted aromatic core due the steric pressure of the *bay*‐substituents. In contrast to the previously studies *ortho*‐substituted TAPPs, these new compounds allowed facile reduction to the monoanionic radical, which was found to be remarkably stable at ambient conditions. Further reduction gave thermally stable dianionic species which were characterized in solution. Both anionic species were characterized by UV/Vis, EPR and NMR spectroscopy, and theoretical modelling. The present work allows for the first time not only to fine‐tune the electronic properties of TAPPs, but simultaneously adjust the geometric properties in a complementary fashion.

## Experimental Section

The syntheses were performed under dried argon in standard Schlenk glassware, which was flame‐dried prior to use. Solvents were dried according to standard procedures. The ^1^H‐,^13^C‐ and ^19^F‐NMR spectra were recorded with Bruker AVANCE 400 and 600II+ spectrometers equipped with variable‐temperature units. ^1^H signals (CDCl_3_: 7.26 ppm, [D_8_]THF: 1.72 ppm/3.58 ppm, [D_6_]DMSO: 2.50 ppm) and ^13^C‐signals (CDCl_3_: 77.16 ppm, [D_8_]THF: 67.21 ppm/25.31 ppm, [D_6_]DMSO: 39.52 ppm) were referenced according to standard literature values.^[64]^ The MALDI mass spectra were obtained from a Bruker apex‐Qe FT‐ICR spectrometer and a Bruker Autoflex Speed MALDI‐TOF spectrometer. The absorption spectra were recorded on a Cary 500 UV/Vis/NIR spectrophotometer and were baseline and solvent corrected. Emission spectra were measured on a Varian Cary Eclipse Spectrophotometer. Description how Fluorescence quantum yields were obtained is explained in detail in the Supporting Information. Cyclic voltammetry was conducted using a standard commercial electrochemical analyser in a three‐electrode single‐component cell under inert atmosphere. The working electrode consists of a platinum disk, the counter electrode of a platinum wire and a saturated calomel electrode was used as a reference electrode. As internal reference ferrocene was used in all cases. As supporting electrolyte 0.1 m tetrabutylammonium hexafluorophosphate was used.

3,4,9,10‐Tetrabromo‐1,6,7,12‐tetrachloroperylene (**1**) was synthesized according to the literature procedures.^1^ All other starting materials were obtained commercially and used without further purification.

### Preparation of compound 2

3,4,9,10‐Tetrabromo‐1,6,7,12‐tetrachloroperylene (2.00 g, 2.83 mmol, 1 equiv.) was suspended in THF (200 mL) and *n‐*butyllithium (7.94 mL, 2.5 m in hexane, 19.8 mmol, 7 equiv.) were added dropwise at −78 °C. After stirring at this temperature for one hour a solution of Tosyl azide (5.59 g, 28.4 mmol, 10 equiv.) in THF (5 mL) was added dropwise and the reaction mixture was allowed to warm up to r.t. overnight. After the addition of H_2_O (30 mL), the phases were separated and the aqueous phase was extracted with DCM (3×100 mL). The united organic phases were dried over Na_2_SO_4_ and evaporation of the solvent gave the crude product, which was further washed with MeOH, acetonitrile and pentane to yield **2** as a red solid (0.75 g, 1.35 mmol, 66 %). ^1^H NMR (600.13 MHz, CDCl_3_ 295 K): *δ* [ppm]=7.35 (s, 4 H). ^13^C NMR (150.90 MHz, CDCl_3_ 295 K): *δ* [ppm]=114.6, 119.4, 122.1, 133.6, 136.6, 136.7.

### Preparation of compound 3

Sodium borohydride (0.221 g, 5.85 mmol, 2.7 equiv.) was added to a solution of **2** (1.20 g, 2.17 mmol, 1 equiv.) in THF (72 mL) and MeOH (6 mL). The reaction mixture was stirred at r.t. for two hours. The reaction was quenched by the addition of 2 m HCl (1.20 mL), stirred for 30 minutes and subsequently aqueous 2 m NaOH (1.20 mL) was added. The solvent was removed in vacuo and the remaining solid was washed with water, MeOH, acetonitrile and pentane and dried in vacuo to give **3** as a red solid (0.75 g, 1.67 mmol, 77 %). ^1^H NMR (600.13 MHz, [D_8_]THF, 295 K): *δ* [ppm]=5.43 (s, 8 H), 6.65 (s, 4 H). ^13^C‐NMR (150.90 MHz, [D_6_]DMSO 295 K): *δ* [ppm]=113.4, 123.5, 125.8, 136.4, 146.5, 159.5. HRMS (MALDI^−^): calcd for C_20_H_12_^35^Cl_3_^37^Cl_1_N_4_ [M]^+⋅^:.449.9787, found: 449.9781.

### Preparation of compound 4

MnO_2_ (3.00 g, 34.5 mmol, 20.7 equiv.) was added to a solution of **3** (0.75 g, 1.67 mmol, 1 equiv) in THF (250 mL). After stirring for one hour at r.t., the reaction mixture was filtered over Celite and the solvent was removed in vacuo to give the product **3** as a green solid (0.72 mg, 1.67 mmol, 96 %).^1^H NMR (600.13 MHz, [D_6_]DMSO, 295 K): *δ* [ppm]=7.30 (s, 4 H), 9.19 (bs, 4 H), 10.48 (bs, 2 H). ^13^C NMR (150.90 MHz, [D_6_]DMSO, 295 K): *δ* [ppm]=101.2, 122.8, 125.0, 134.6, 158.2. HRMS (MALDI^−^): calcd for C_20_H_10_^35^Cl_3_^37^Cl_1_N_4_ [*M*]^+⋅^: 447.9630; found: 447.9656.

### Preparation of compound 5 a

Triethylamine (435 μL, 3.12 mmol, 2.8 equiv.) and trifluoroacetic anhydride (374 μL, 2.79 mmol, 2.5 equiv.) were added to a solution of DPDI‐Cl (**4**) (500 mg, 1.12 mmol, 1 equiv.) in THF (10 mL) and the reaction mixture was refluxed for 72 h. After cooling to r.t., the volatiles were removed in vacuo and the crude mixture was separated by column chromatography (petroleum ether/ethyl acetate 10:1) to give the product **5 a** as a yellow solid (75 mg, 124 μmol, 11 %). ^1^H NMR (600.13 MHz, CDCl_3_ 295 K): *δ* [ppm]=8.74 (s, 4 H). ^13^C NMR (150.90 MHz, CDCl_3_ 295 K): *δ* [ppm]=112.7, 125.3, 126.1, 130.7, 141.1, 153.8. Residual ^13^C signals of N‐**C**‐N‐ and **C**F_3_‐groups could not be detected due to coupling to fluorine groups and low solubility. ^19^F NMR (376.27 MHz, CDCl_3_ 295 K): *δ* [ppm]=−69.0 (s, 6 F). HRMS (MALDI^−^): calcd for C_24_H_4_^35^Cl_3_^37^Cl_1_F_6_N_4_ [*M*]^−⋅^: 603.9065; found: 603.9059. Redox potentials measured against SCE in THF: *E*_red1_=−0.18 V, *E*_red2_=−0.49 V.

### Preparation of compound 5 b:

Triethylamine (414 μL, 2.97 mmol, 2.8 equiv.) and pentafluoropropionic anhydride (526 μL, 2.66 mmol, 2.5 equiv) were added to a solution of DPDI‐Cl (**4**) (476 mg, 1.06 mmol, 1 equiv.) in dioxane (10 mL) and the solution was refluxed for 72 h. After cooling to r.t., the solvent was removed in vacuo and the residue was washed thoroughly with water (200 mL). The crude compound was recrystallized in hot ethanol to afford **5 b** as a yellow solid (170 mg, 241 μmol, 23 %).^1^H‐NMR (600.13 MHz, CDCl_3_ 295 K): *δ* [ppm]=8.76 (s, 4 H). ^13^C NMR (150.90 MHz, CDCl_3_ 295 K): *δ* [ppm]=112.6, 125.3, 126.1, 130.8, 141.1, 153.8, 156.1. Residual ^13^C‐signals of the perfluorinated pentyl groups could not be detected due to coupling to fluorine groups. ^19^F NMR (376.27 MHz, CDCl_3_ 295 K): *δ* [ppm]=−81.4 (t, ^3^ *J*_F−F_=1.75 Hz, 6 F), −116.0 (bs, 4 F). HRMS (MALDI^−^): calcd for C_26_H_4_^35^Cl_3_^37^Cl_1_F_10_N_4_ [*M*]^−⋅^: 703.9001; found: 703.9006. Redox potentials measured against SCE in THF: *E*_red1_=−0.18 V, *E*_red2_=−0.49 V.

### Preparation of compound 5 c:

Triethylamine (217 μmol, 1.56 mmol, 2.8 equiv.) and perfluorobutyryl chloride (207 μl, 1.39 mmol, 2.5 equiv) were added to a solution of DPDI‐Cl (**4**) (250 mg, 0.56 mmol, 1 equiv.) in THF (10 mL) and the solution was refluxed for 72 h. After cooling to r.t., the solvent was removed in vacuo and the residue was washed thoroughly with water (200 mL) and the crude product purified by column chromatography (petroleum ether/ethyl acetate 10:1) to give **5 c** as a yellow solid (54 mg, 76 μmol, 14 %). ^1^H‐NMR (600.13 MHz, CDCl_3_ 295 K): *δ* [ppm]=8.77 (s, 4 H). ^13^C‐NMR (150.90 MHz, CDCl_3_ 295 K): *δ* [ppm]=112.5, 125.3, 126.1, 130.8, 141.1, 153.7. Residual ^13^C‐signals of the N‐**C**‐N‐ and perfluorinated propyl‐groups could not be detected due to coupling to fluorine groups. ^19^F NMR (376.27 MHz, CDCl_3_ 295 K): *δ* [ppm]=−80.1 (t, ^3^ *J*_F−F_=9.03 Hz, 6 F), −114.0–114.1 (m, 4 F), −125.4 (bs, 4 F). HRMS (MALDI^−^): calcd for C_28_H_4_^35^Cl_3_^37^Cl_1_F_14_N_4_ [*M*]^−⋅^: 803.8937; found: 803.8932. Redox potentials measured against SCE in THF: *E*_red1_=−0.19 V, *E*_red2_=−0.49 V.

### Preparation of compound 5 d:

DPDI‐Cl (**4**) (100 mg, 0.223 mmol, 1 equiv) was dissolved in 5 mL triethylorthoformiate and 1 mL of formic acid was added. The solution was stirred for 3 d at 110 °C. After cooling to r.t., the volatiles were removed in vacuo and residue was washed with water, MeOH and pentane. The pure compound **5 d** was isolated as a yellow powder (25 mg, 0.053 mmol, 24 %). ^1^H NMR (600.13 MHz, CDCl_3_ 295 K): *δ* [ppm]=10.01 (s, 2 H), 8.59 (s, 4 H). ^13^C NMR could not be measured due to low solubility. HRMS (MALDI^−^): calcd for C_22_H_6_^35^Cl_3_^37^Cl_1_N_4_ [*M*]^−⋅^: 467.9317; found: 467.9315. Redox potentials measured against SCE in THF: *E*_red1_=−0.383 V, *E*_red2_=−0.681 V.

### Theoretical methods

Frontier orbitals and electron affinities: The DFT calculations to obtain orbital energies and electron affinities were carried out using ORCA 4.0.1 program package.^[65]^ B3LYP was employed as functional;[^66^, ^67^] a def2‐SVP basis set was used for all atoms during geometry optimizations.[^68^, ^69^] Every optimization was verified by frequency calculations. All other properties were calculated using a def2‐QZVPP basis set.^[70]^

Vertical transitions: Calculations of the vertical excitation energies (VEE) and vertical de‐excitation energies (VDE), employing B3LYP together with the def2‐TZVPP basis, have been carried out using the Turbomole program package V7.2[^71^, ^72^] using default convergence criteria for both the response equations and the geometry convergence. Accurate geometries for the Franck‐Condon simulations were obtained with tightened thresholds of 10^−9^ and employing *D*_2_ symmetry.^[48]^ The calculations of the vibronic (Franck–Condon) spectra were conducted using the HOTFCHT program.[^73^, ^74^, ^75^]

Interconversion energies: The geometries and their relative energies have been computed using the Turbomole program V7.2. PBE0 was employed as functional together with def2‐TZVP basis and the RI approximation (RI‐J). The stationary points were verified with analytical second derivatives.

## Conflict of interest

The authors declare no conflict of interest.

## Supporting information

As a service to our authors and readers, this journal provides supporting information supplied by the authors. Such materials are peer reviewed and may be re‐organized for online delivery, but are not copy‐edited or typeset. Technical support issues arising from supporting information (other than missing files) should be addressed to the authors.

Supplementary
