# Supplementary material for: Twisting the TAPPs: Bay‐Substituted Non‐planar Tetraazapero‐pyrenes and their Reduced Anions
Source: Chemistry. 2019 Oct 22;25(64):14669–78. doi: 10.1002/chem.201903413 (PMC7687186; doi:10.1002/chem.201903413)
Supplement: Supplementary file 1 — Supplementary [file CHEM-25-14669-s001.pdf]

CHEMISTRY
A European Journal

Supporting Information

Twisting the TAPPs: *Bay*-Substituted Non-planar Tetraazaperopyrenes and their Reduced Anions

Benjamin A. R. Günther,[a] Sebastian Höfener,[b] Ute Zschieschang,[c] Hubert Wadepohl,[a] Hagen Klauk,[c] and Lutz H. Gade*[a]

chem_201903413_sm_miscellaneous_information.pdf

Table of Contents

The ^{1}H, ^{13}C and ^{19}F NMR Spectra of Compounds S2

Absorption Spectra of Compounds 5a-d S11

Emission Spectra of Compounds 5a-d S13

Cyclic Voltammograms of Compounds 5a-d S16

Characterization of Reduced Species S18

^{1}H and ^{19}F-NMR-Spectra of $5a^{2-}$ S19

Absorption Spectra of 5a, $5a^{\cdot-}$ and $5a^{2-}$ S20

Computational Methods. S21

TFT Fabrication Process S31

Crystal Structures of Compounds 5a-d S31

References S35

The ^{1}H, ^{13}C and ^{19}F NMR Spectra of Compounds

Compound 2

^{1}H-NMR (600.13 MHz, $CDCl_3$, 295 K):

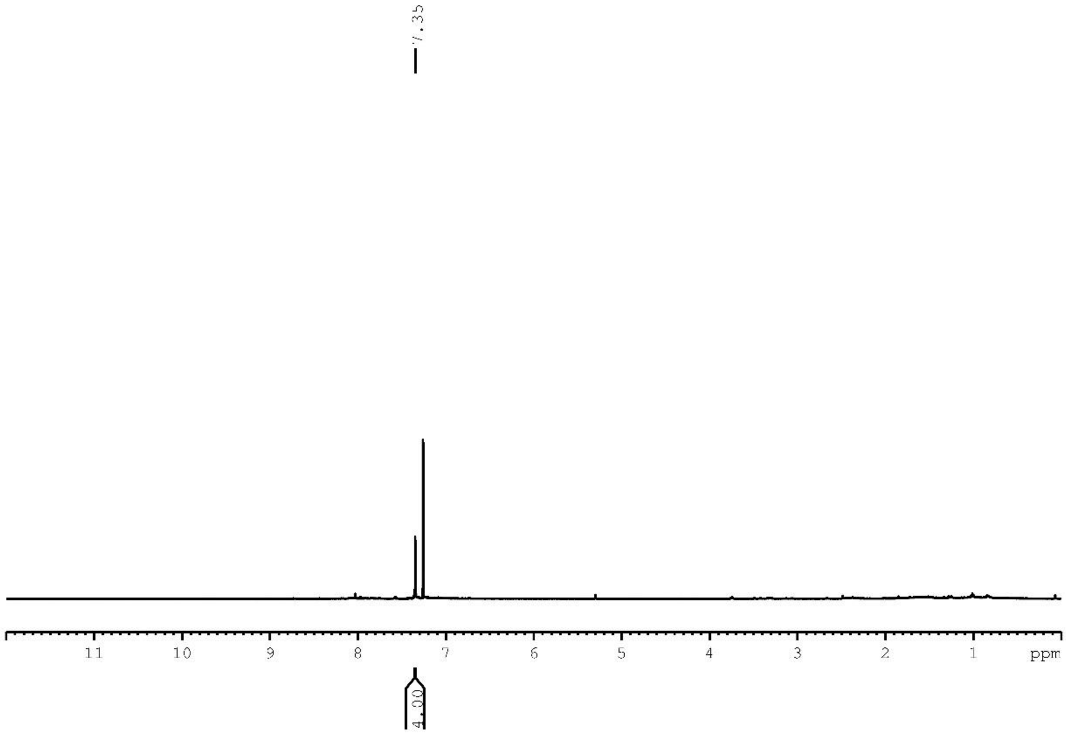

^{13}C-NMR (150.90 MHz, $CDCl_3$, 295 K):

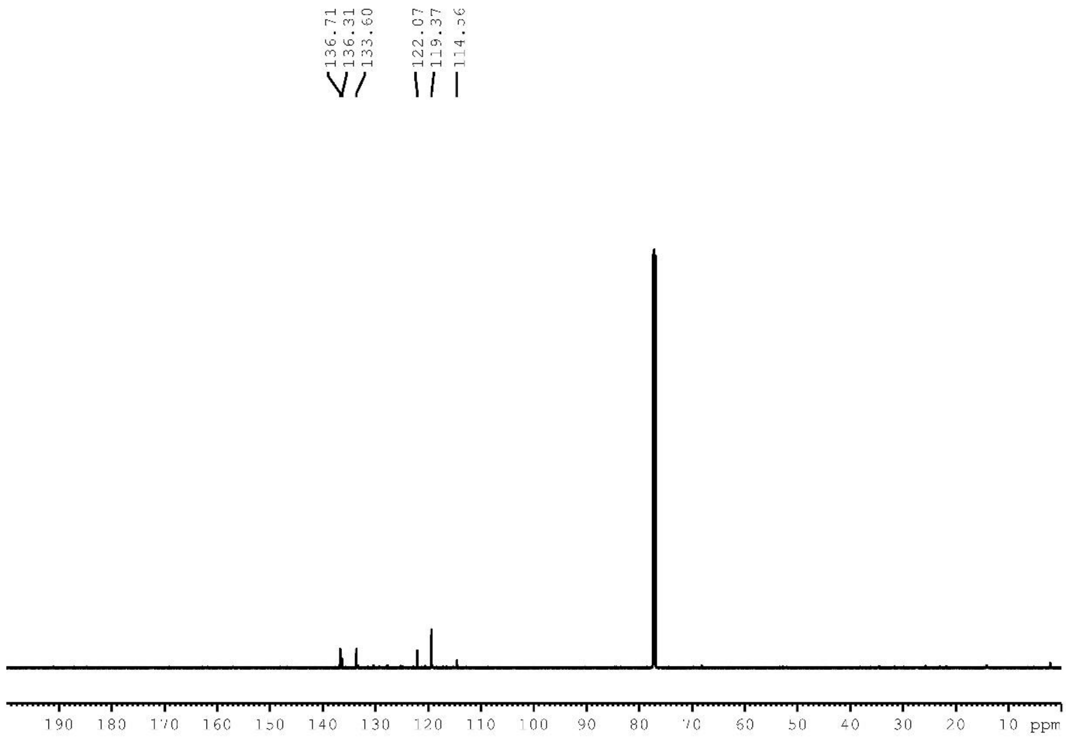

Compound 3

^{1}H-NMR (600.13 MHz, thf-d_8, 295 K):

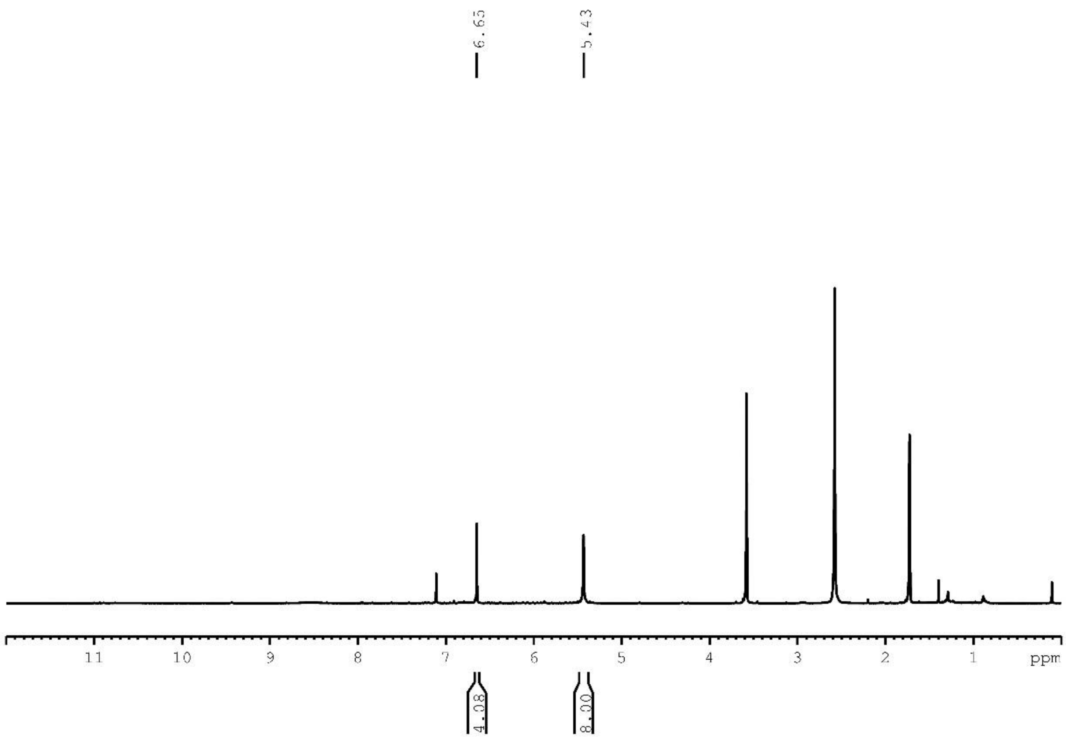

^{13}C-NMR (150.90 MHz, dmso-d_6, 295 K):

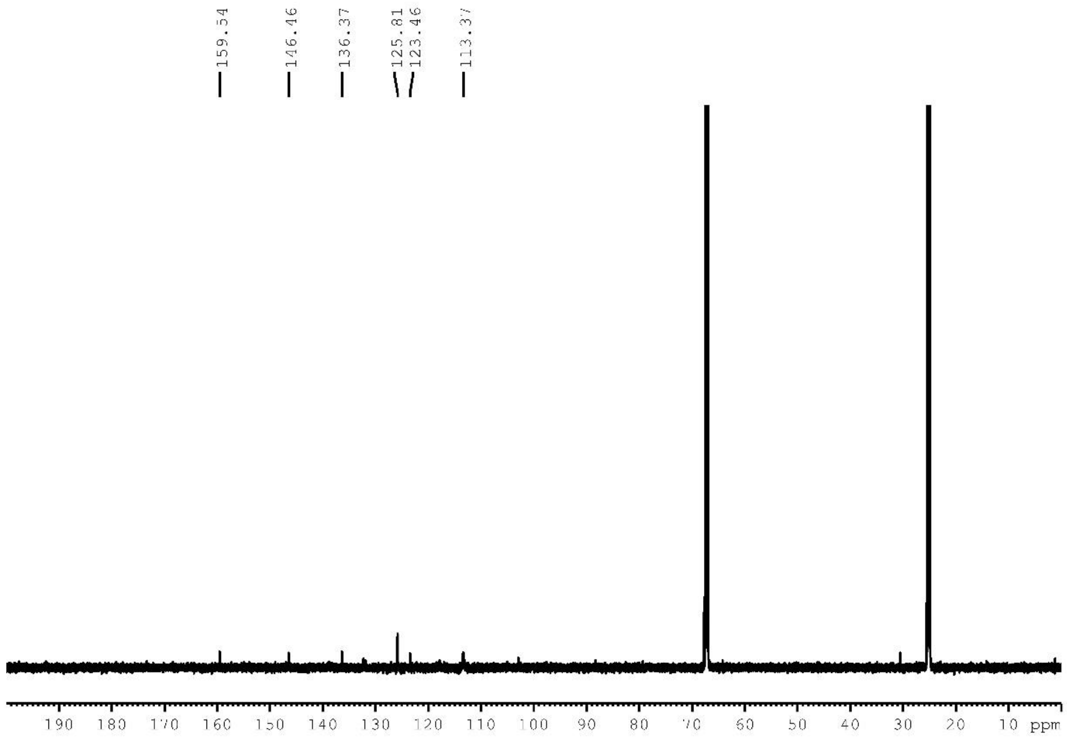

Compound 4

^{1}H-NMR (600.13 MHz, dmso-d_6, 295 K):

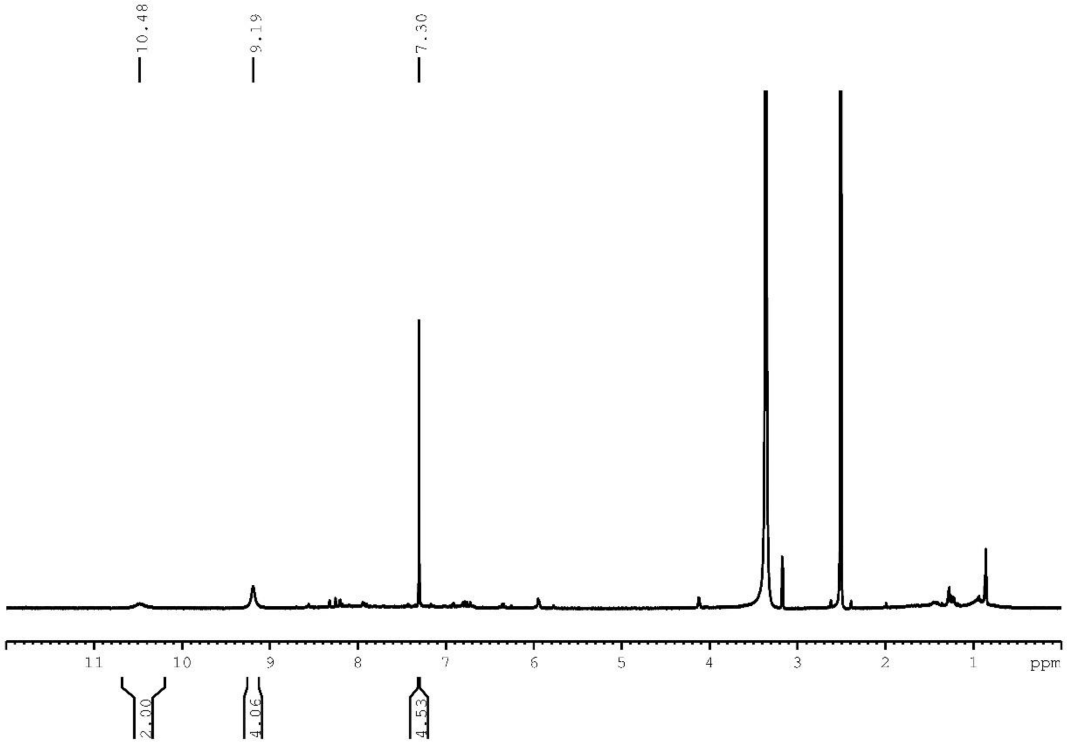

^{13}C-NMR (150.90 MHz, dmso-d_6, 295 K):

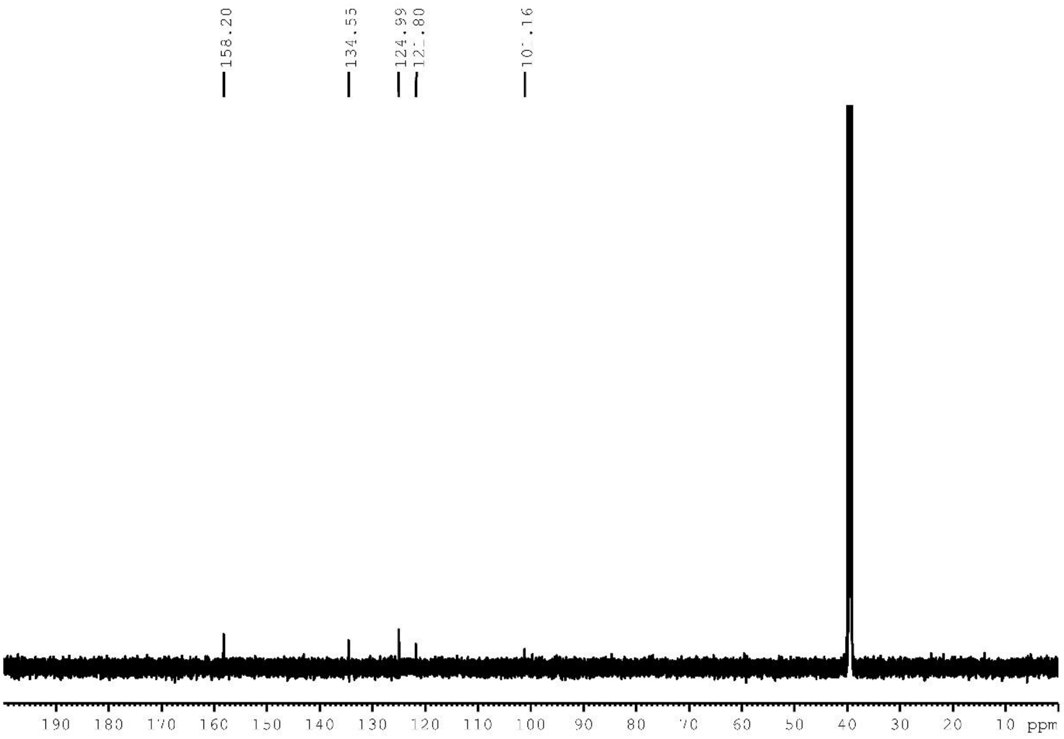

Compound 5a (CF_3-TAPP-Cl_4)

^{1}H-NMR (600.13 MHz, $CDCl_3$, 295 K):

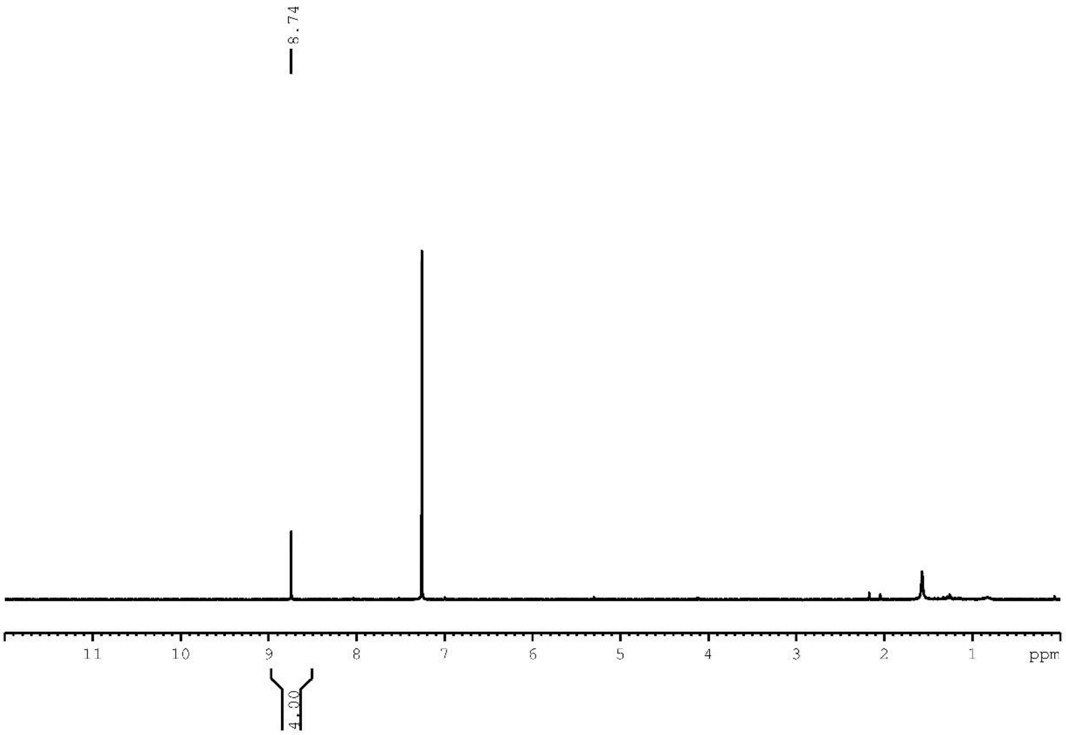

^{13}C-NMR (150.90 MHz, $CDCl_3$, 295 K):

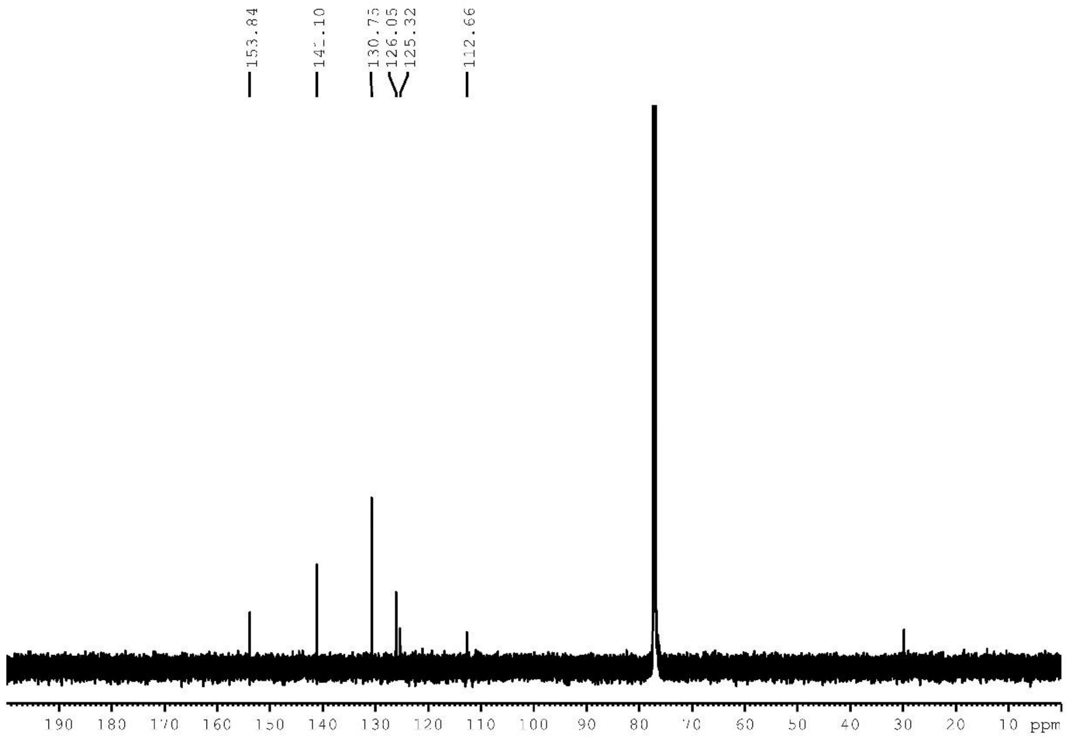

^{19}F-NMR (376.27 MHz, $CDCl_3$, 295 K):

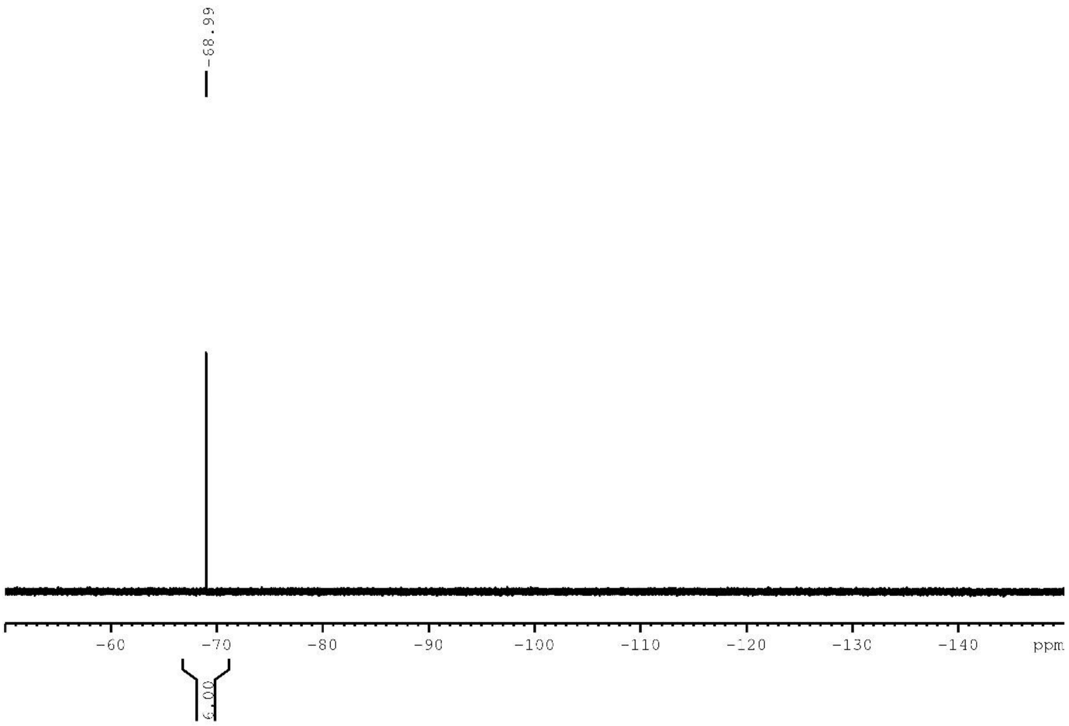

Compound 5b (C_2F_5-TAPP-Cl_4)

^{1}H-NMR (600.13 MHz, $CDCl_3$, 295 K):

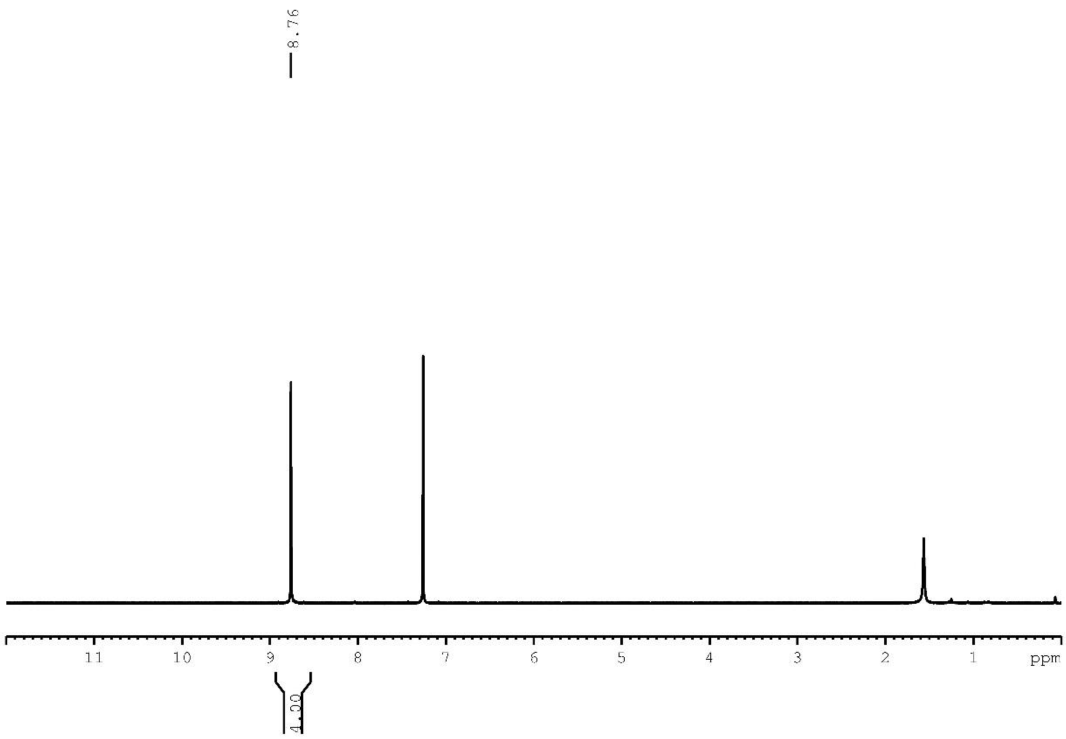

^{13}C-NMR (150.90 MHz, $CDCl_3$, 295 K):

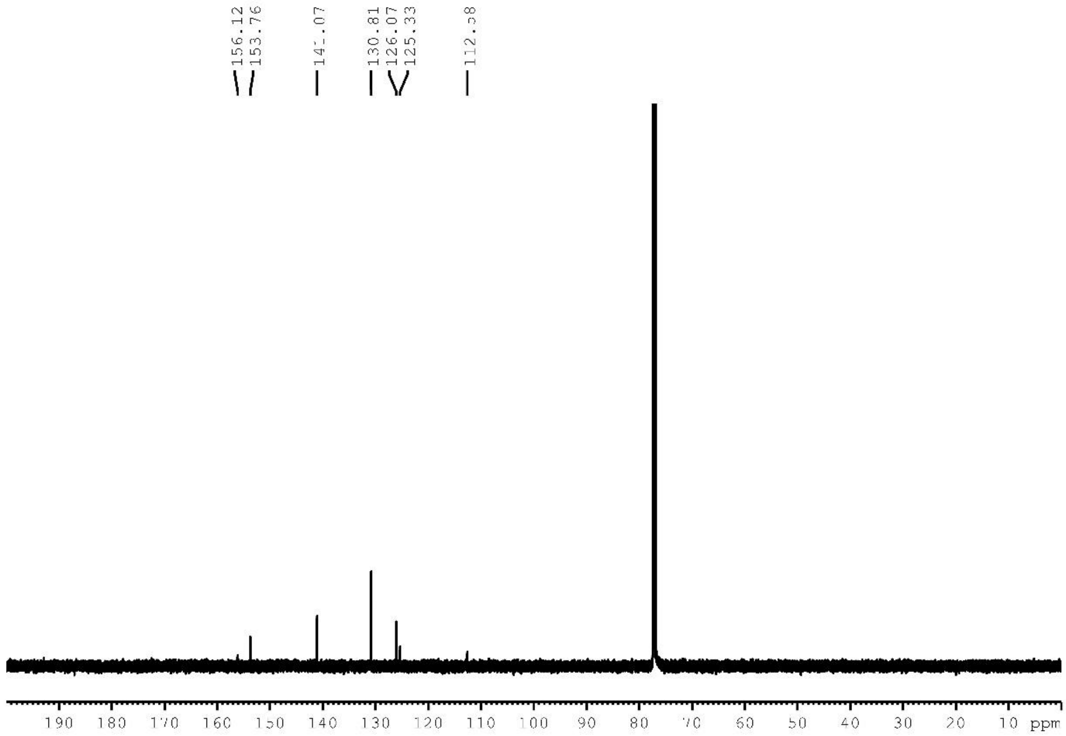

^{19}F-NMR (376.27 MHz, $CDCl_3$, 295 K):

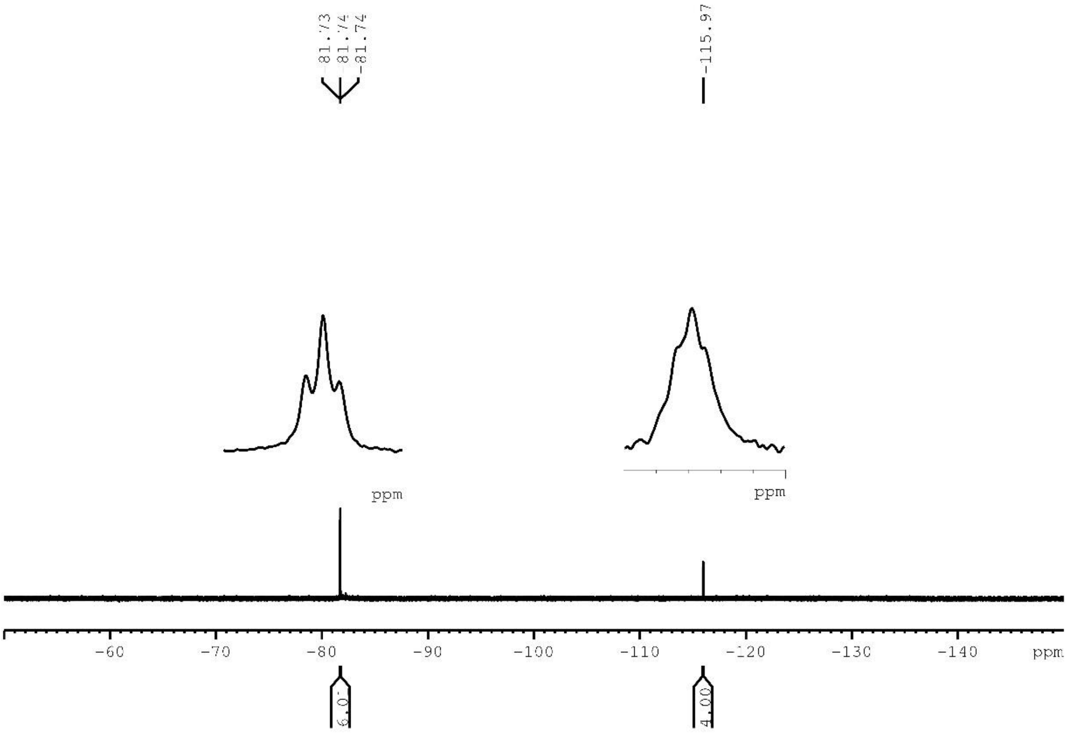

Compound 5c (C_3F_7-TAPP-Cl_4)

^{1}H-NMR (600.13 MHz, $CDCl_3$, 295 K):

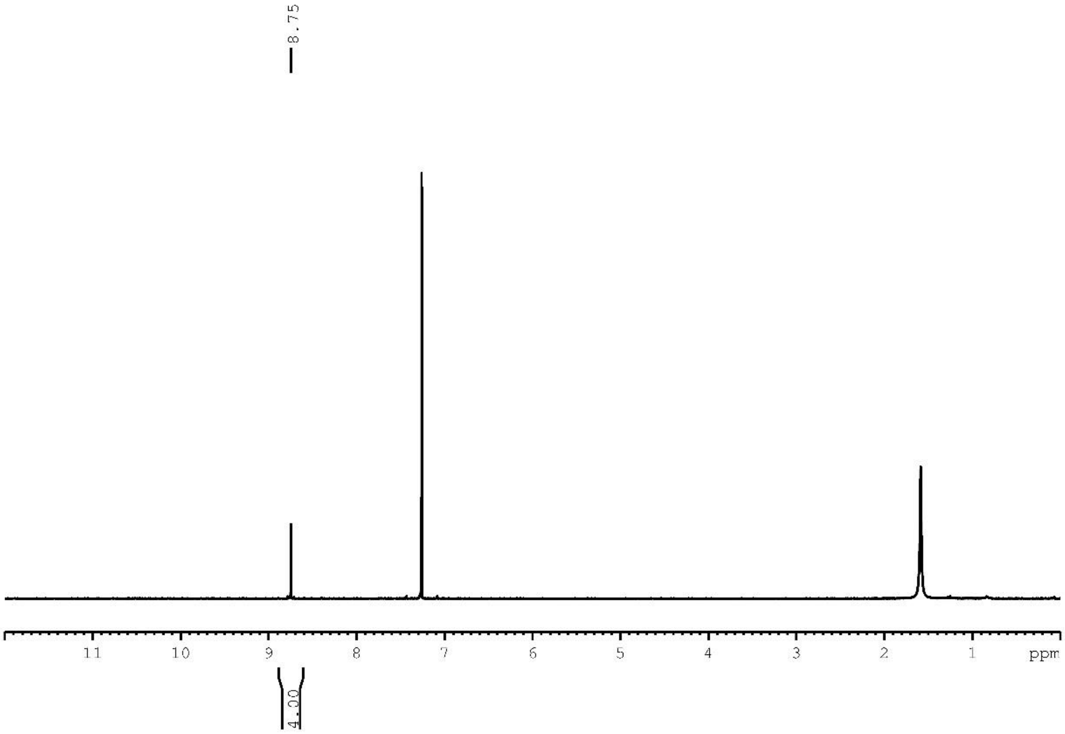

^{13}C-NMR (150.90 MHz, $CDCl_3$, 295 K):

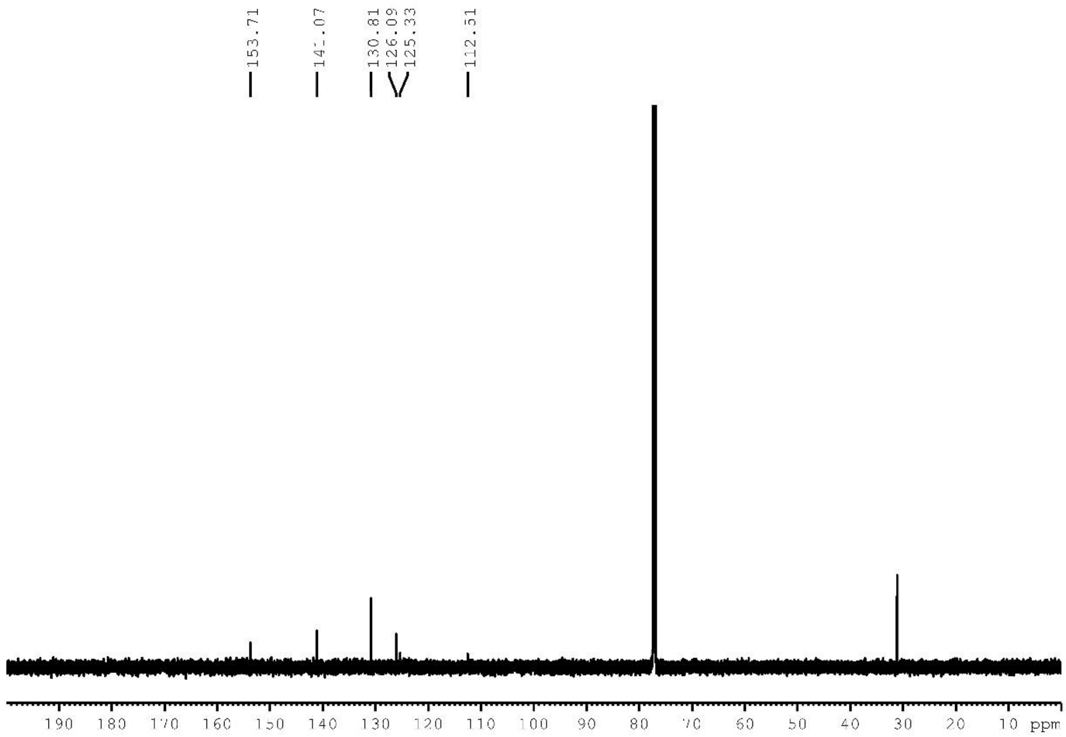

^{19}F-NMR (376.27 MHz, $CDCl_3$, 295 K):

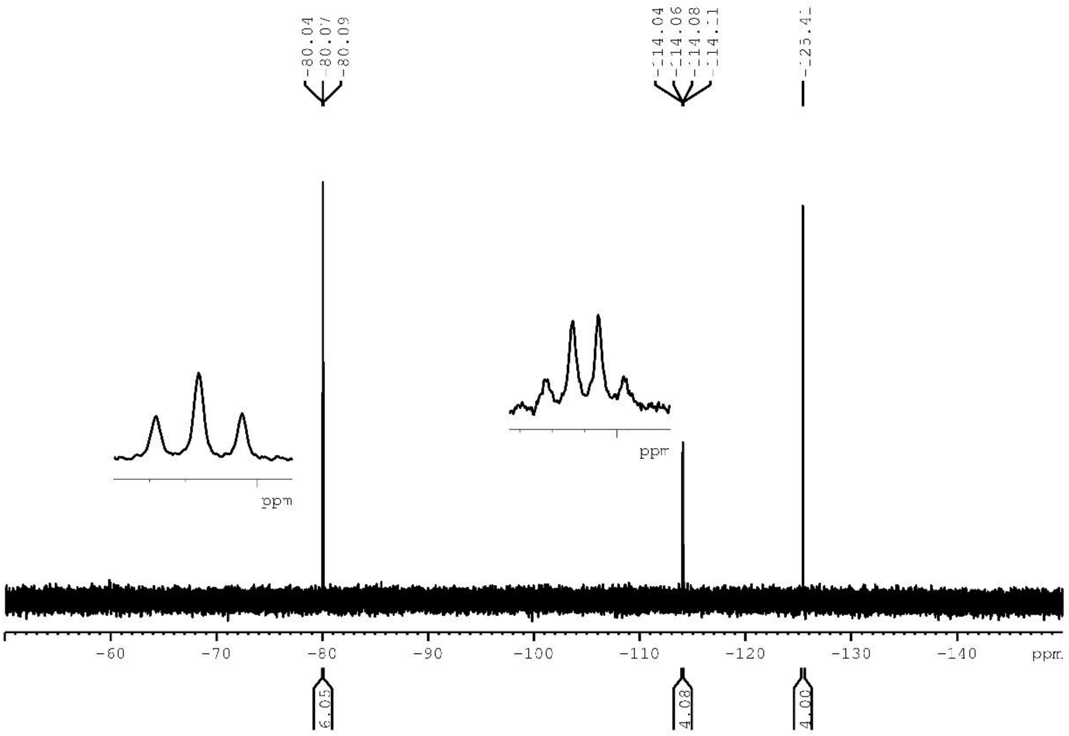

Compound 5d (H-TAPP-Cl_4)

^{1}H-NMR (600.13 MHz, $CDCl_3$, 295 K):

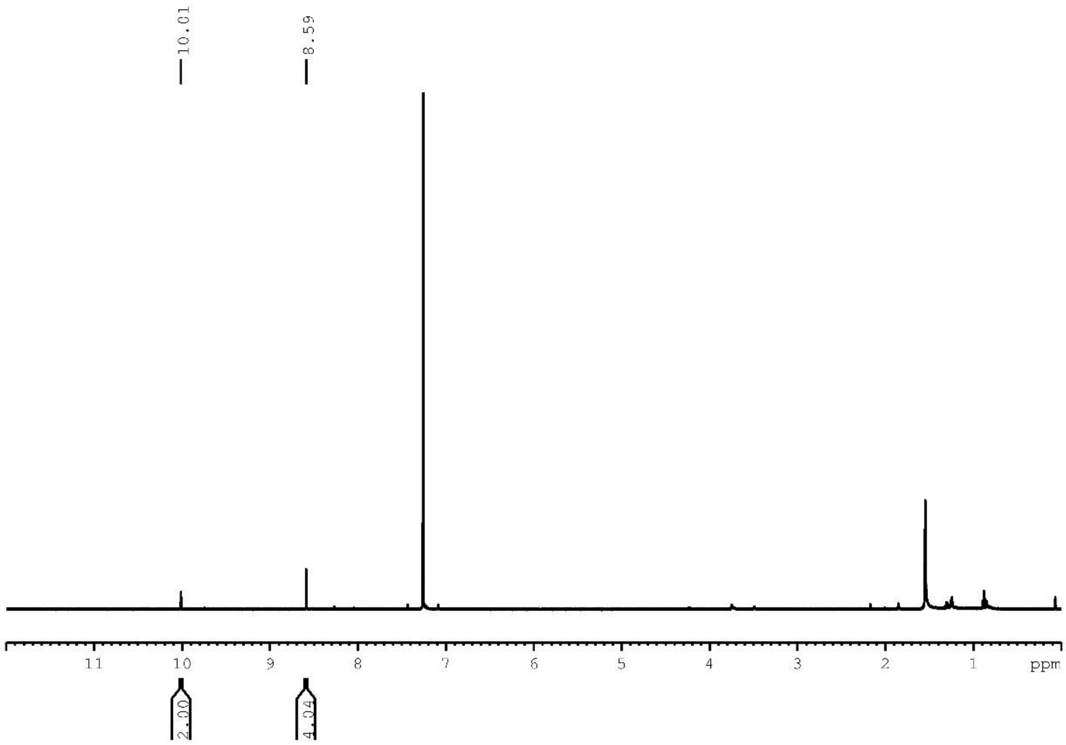

Absorption Spectra of Compounds 5a-d

Compound 5a (CF_3-TAPP-Cl_4)

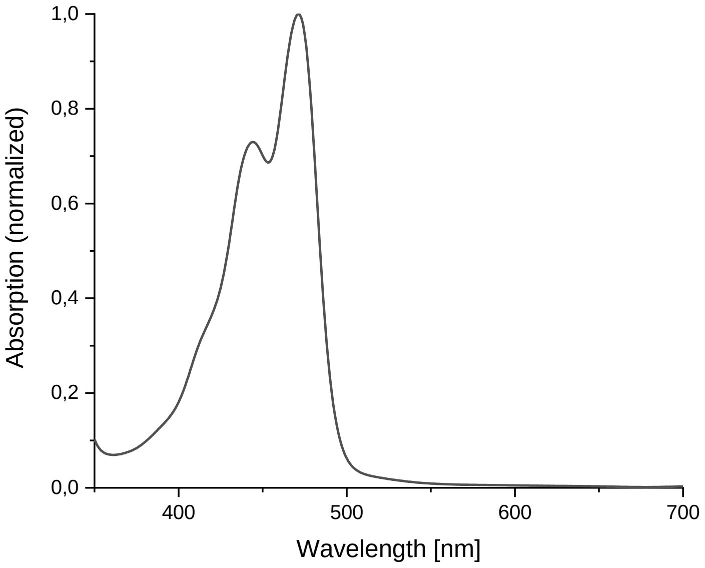

Compound 5b (C_2F_5-TAPP-Cl_4)

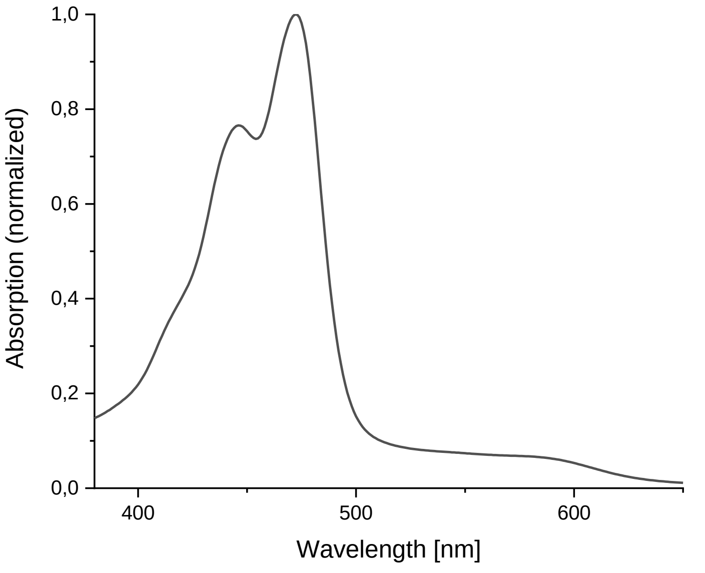

Compound 5c (C_3F_7-TAPP-Cl_4)

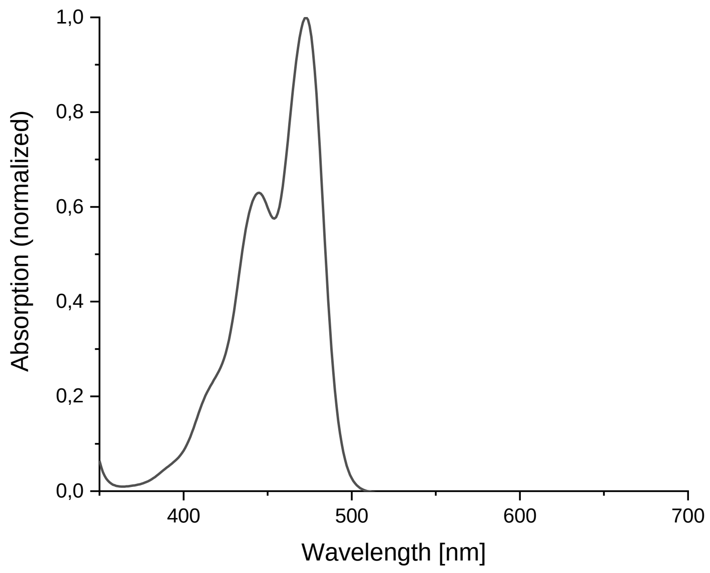

Compound 5d (H-TAPP-Cl_4)

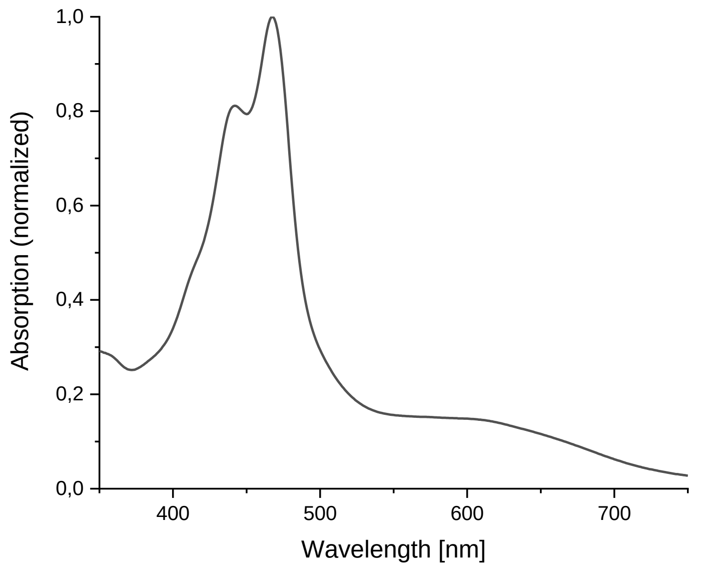

Emission Spectra of Compounds 5a-d

Fluorescence quantum yields were determined in dilute solutions (OD < 1 at the excitation wavelength) and referenced to Fluorescin by using equation (**1**). A is the absorbance at the excitation wavelength (λ), I is the intensity of the excitation light at (λ), n is the refractive index of the solvent, D is the integrated scripts and r and x refer to the reference and sample. All quantum yields were obtained at identical excitation wavelengths of sample and reference in order to cancel out the term $I(\lambda_r)/I(\lambda_x)$ of equation (**1**).

$$\textbf{(1)}\ \boldsymbol{\Phi_x = \Phi_r\, \left(\frac{A_r(\lambda_r)}{A_x(\lambda_x)}\right)\left(\frac{I_r(\lambda_r)}{I_x(\lambda_x)}\right)\left(\frac{n_x^2}{n_r^2}\right)\left(\frac{D_x}{D_r}\right)}$$

Compound 5a (CF_3-TAPP-Cl_4)

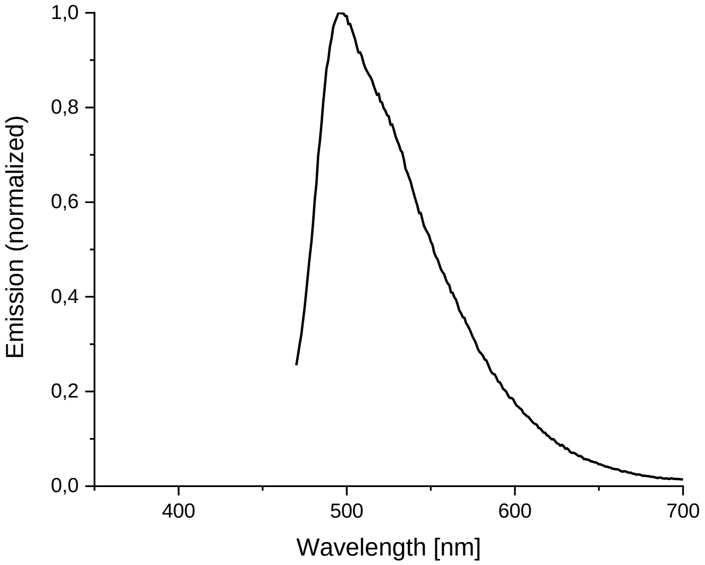

Compound 5b (C_2F_5-TAPP-Cl_4)

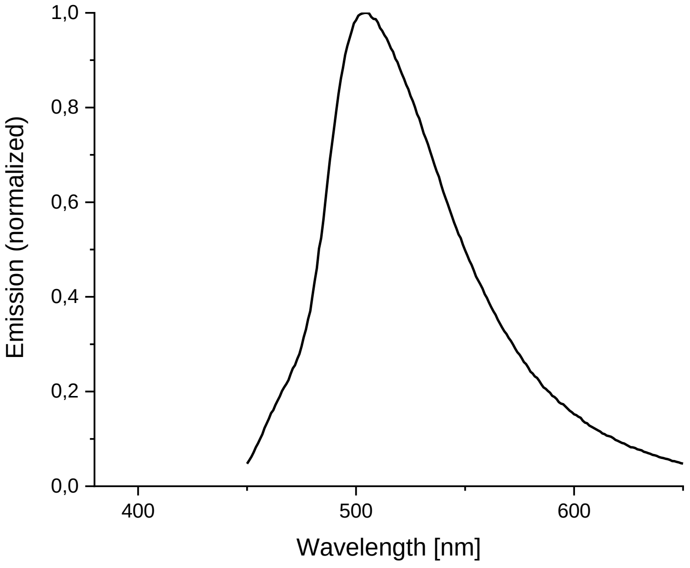

Compound 5c (C_3F_7-TAPP-Cl_4)

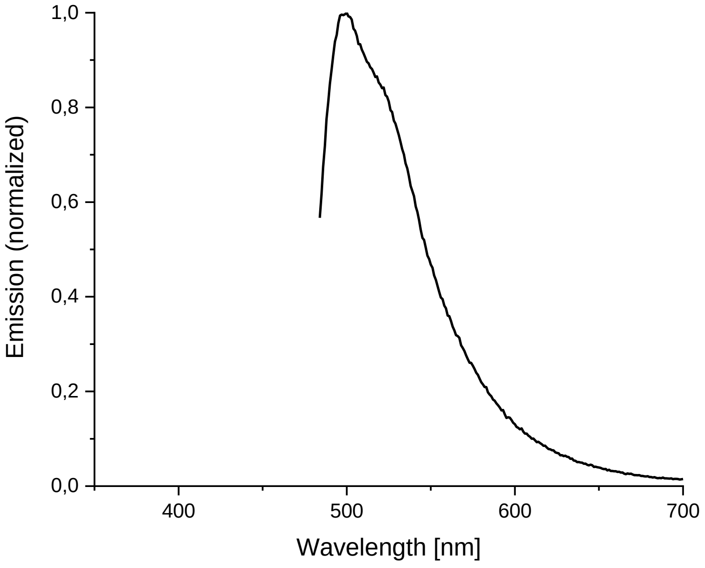

Compound 5d (H-TAPP-Cl_4)

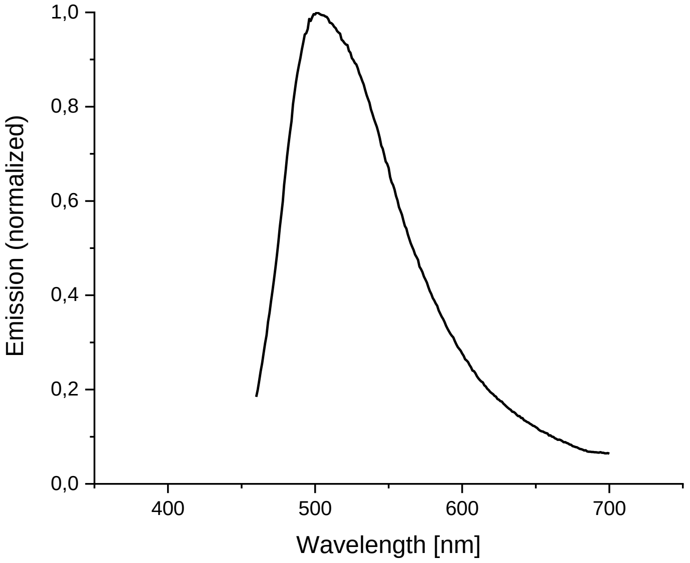

Cyclic Voltammograms of Compounds 5a-d

Compound 5a (CF_3-TAPP-Cl_4)

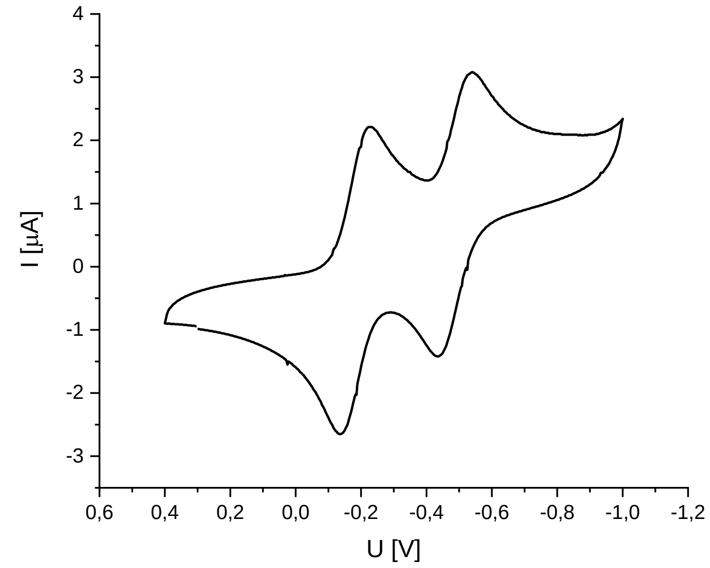

Compound 5b (C_2F_5-TAPP-Cl_4)

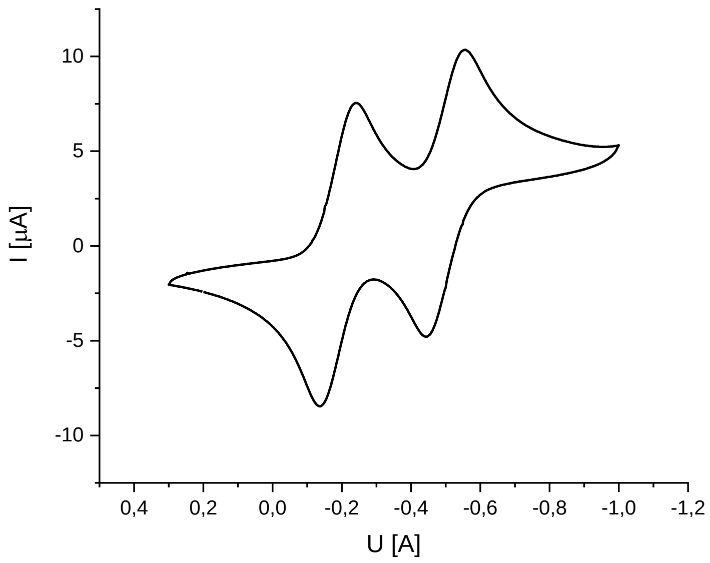

Compound 5c (C_3F_7-TAPP-Cl_4)

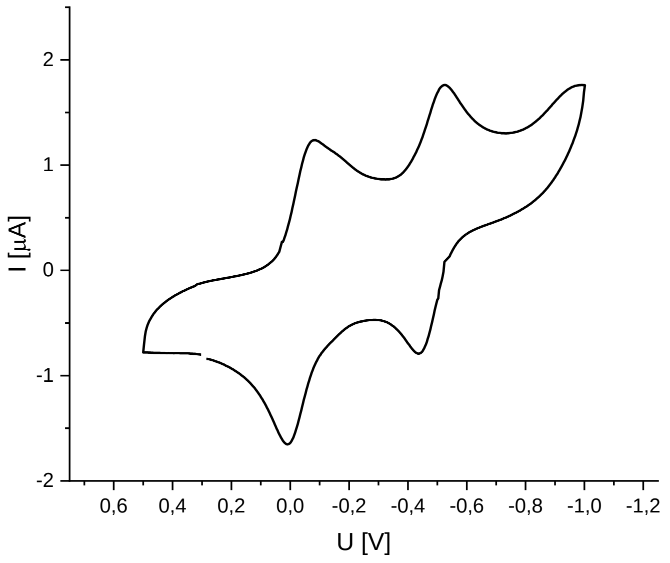

Compound 5d (H-TAPP-Cl_4)

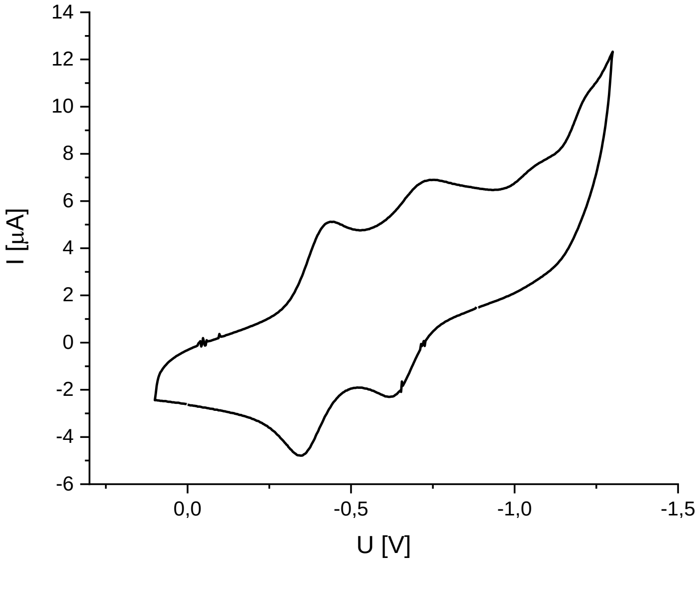

Characterization of Reduced Species

ESR-Spectra of Compounds 5a-c

Compound 5a (CF_3-TAPP-Cl_4)

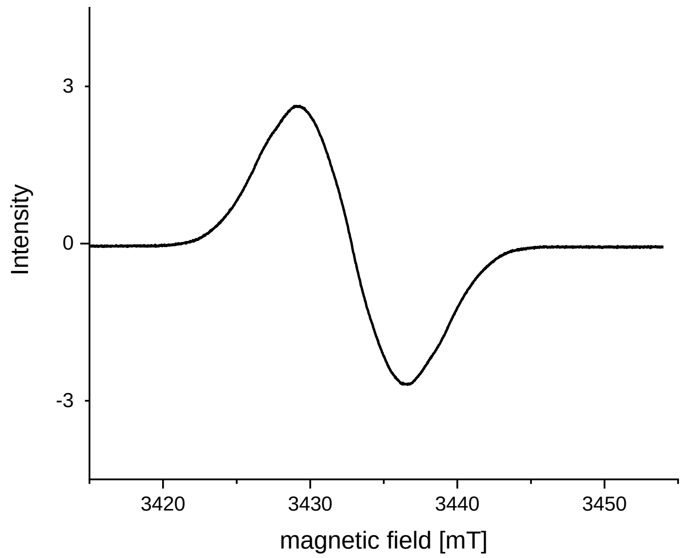

Compound 5b (C_2F_5-TAPP-Cl_4)

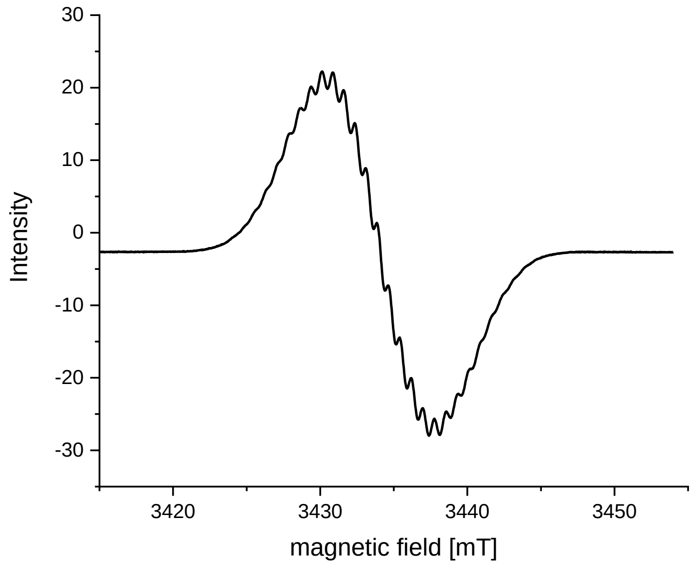

Compound 5c (C_3F_7-TAPP-Cl_4)

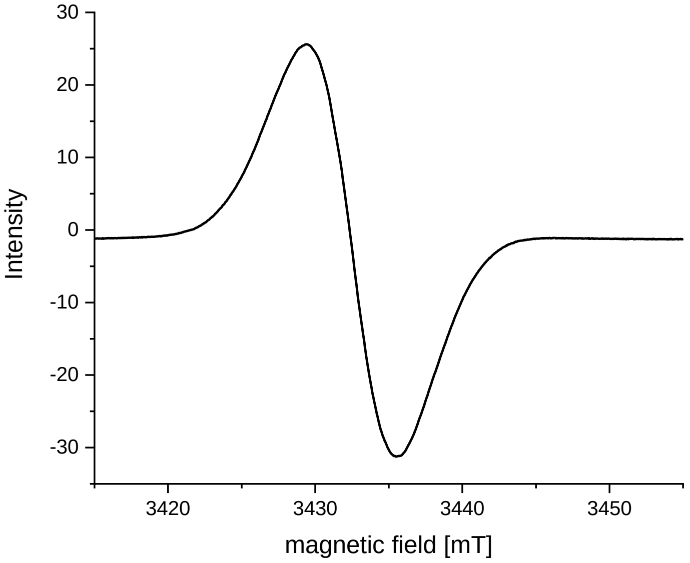

^{1}H and ^{19}F-NMR-Spectra of $5a^{2-}$

^{1}H-NMR (600.13 MHz, $CDCl_3$, 295 K):

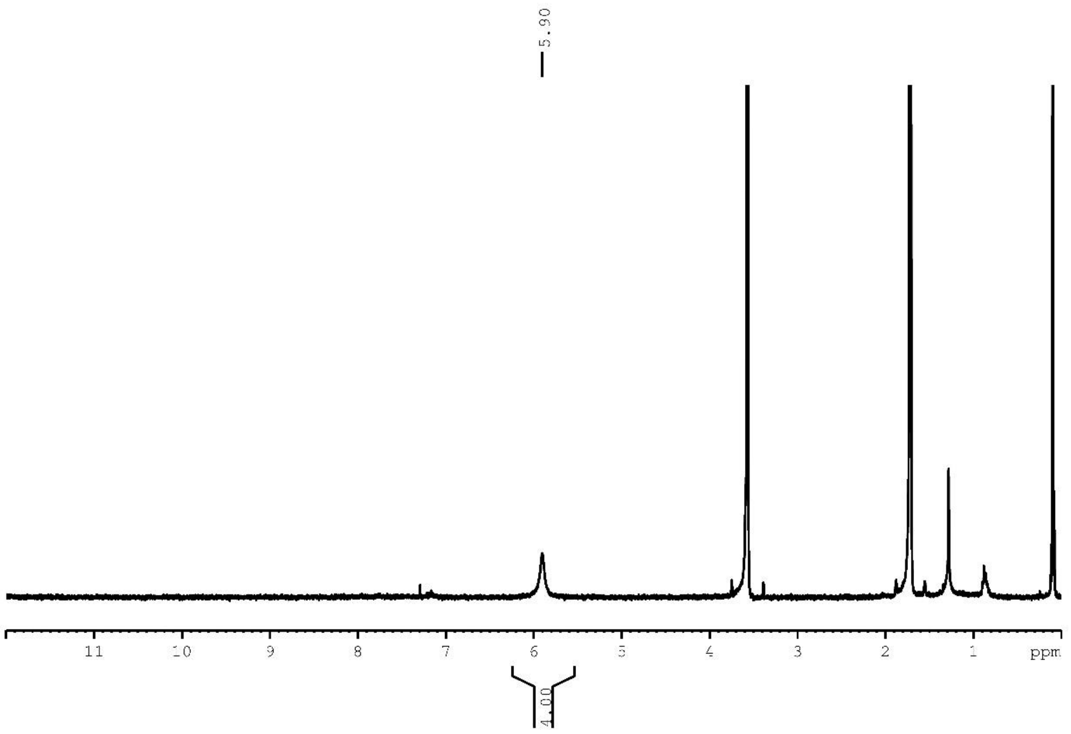

^{19}F-NMR (376.27 MHz, $CDCl_3$, 295 K):

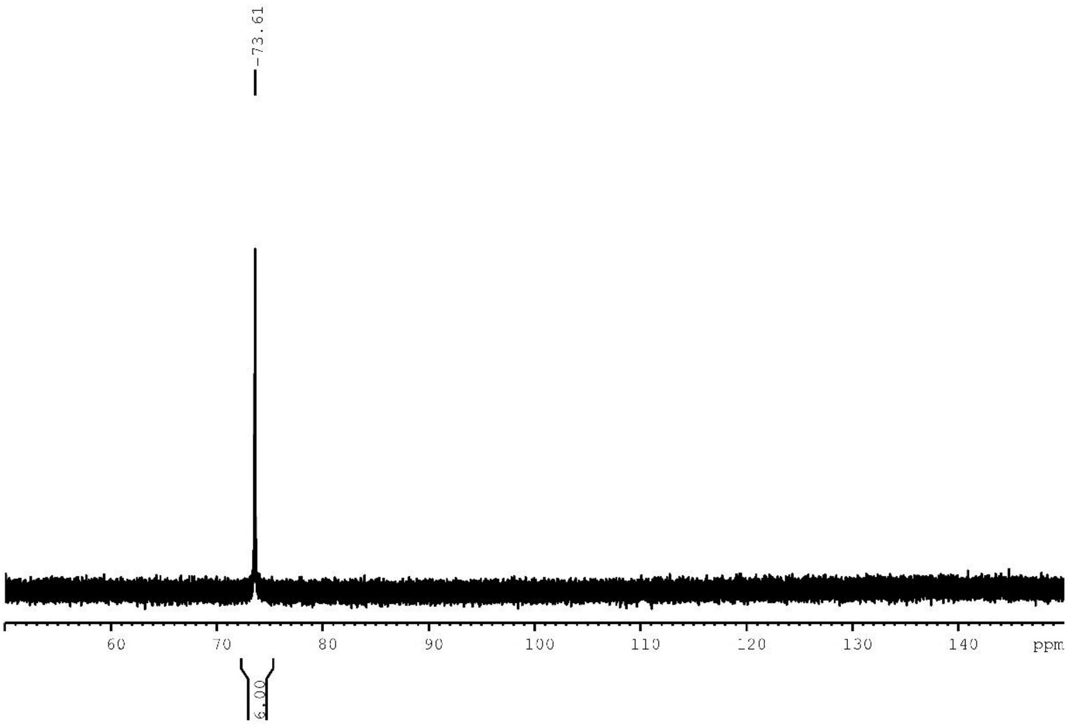

Absorption Spectra of 5a, 5a$^{\cdot-}$ and 5a^{2-}

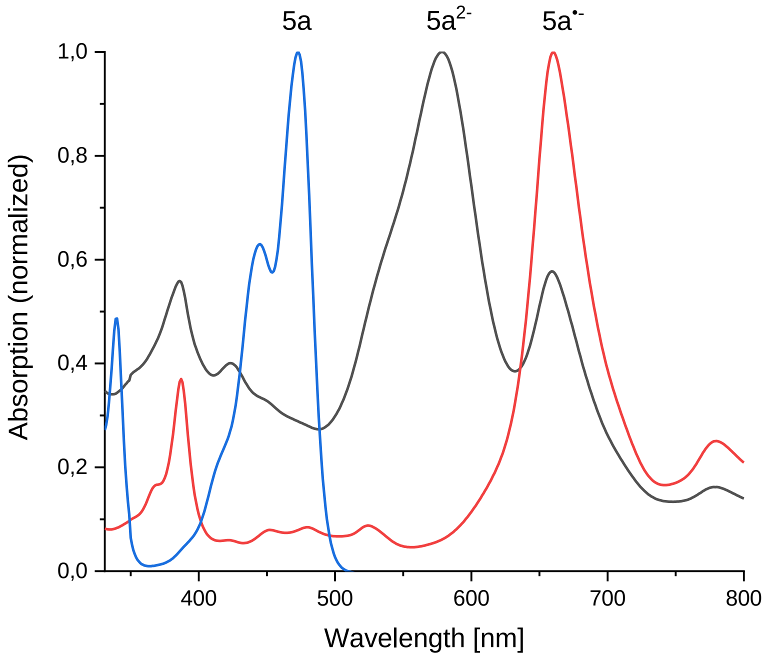

Computational Methods.

Coordinates of Compound 5a (CF_3-TAPP-Cl_4)

Atomic Type	Coordinates [Å]		
	X	Y	Z
F	12.325717	9.972215	6.177854
F	13.588718	8.208711	6.170118
N	11.89735	8.918009	3.207875
N	14.141972	9.459602	3.822909
C	12.186645	9.130178	1.91354
C	11.165018	9.049499	0.912535
C	11.494489	9.164018	-0.41096
C	12.871626	9.303096	-0.85857
C	13.829384	9.691001	0.135888
C	13.491172	9.519844	1.508627
C	14.451716	9.700803	2.537211
C	15.787661	10.050507	2.162364
C	16.108134	10.270184	0.848039
C	13.364626	9.096085	-2.179707
C	12.895941	9.069868	4.072941
C	12.567808	8.809554	5.547253
N	16.462514	10.563853	-5.644494
N	14.810772	8.908884	-6.136989
C	16.045259	10.621293	-4.367938
C	16.618368	11.557203	-3.444644
C	16.250379	11.531307	-2.124735
C	15.329997	10.538514	-1.591405
C	14.545158	9.820819	-2.553905
C	14.969652	9.811005	-3.913503
C	14.353416	8.968602	-4.874803
C	13.292703	8.107799	-4.448847
C	12.837731	8.157526	-3.157837
C	15.119398	10.211612	-0.218041
C	15.833642	9.701257	-6.439323
F	11.488708	8.037425	5.681085
C	16.408267	9.52699	-7.851186
F	16.937587	10.6582	-8.322384
F	17.38503	8.601376	-7.822347
F	15.475452	9.110546	-8.712182
H	17.302691	12.316996	-3.823912
H	16.555587	10.062573	2.936529

H	10.127213	8.944265	1.230793
H	12.906861	7.370179	-5.153032
Cl	16.738785	12.870873	-1.124545
Cl	17.80613	10.382171	0.467317
Cl	10.19415	9.366004	-1.55294
Cl	11.758487	6.88322	-2.661287

Final Single Point Energy	-3575.65818245572 E_h	
HOMO:	-0.251282 E_h	-6.8377 eV
LUMO:	-0.151553 E_h	-4.1240 eV

Coordinates of Compound 5a⁻ (CF_3-TAPP-Cl_4)

Atomic Type	Coordinates [Å]		
	X	Y	Z
F	12.236489	10.115348	6.164239
F	13.510945	8.363829	6.241052
N	11.8498	9.006571	3.232876
N	14.11934	9.46584	3.861496
C	12.161484	9.178881	1.922636
C	11.169929	9.099639	0.912183
C	11.538419	9.151013	-0.422133
C	12.897744	9.253329	-0.85034
C	13.848978	9.634751	0.156176
C	13.493116	9.498913	1.528235
C	14.459969	9.659011	2.561441
C	15.796098	9.944011	2.187018
C	16.118943	10.152312	0.855719
C	13.419335	9.017389	-2.182262
C	12.853959	9.14051	4.090182
C	12.502238	8.934787	5.563334
N	16.425544	10.625297	-5.681588
N	14.822485	8.909136	-6.174183
C	16.035868	10.640114	-4.380644
C	16.597932	11.551197	-3.449124
C	16.252095	11.471513	-2.109063
C	15.363956	10.48019	-1.588928
C	14.589384	9.759497	-2.560835
C	14.998457	9.774568	-3.924799
C	14.392983	8.915764	-4.886351
C	13.38365	8.022951	-4.448614
C	12.934445	8.073015	-3.139444
C	15.151449	10.128495	-0.196665
C	15.796793	9.759262	-6.467909

F	11.424213	8.156754	5.724418
C	16.328575	9.664793	-7.898639
F	16.759131	10.843958	-8.368205
F	17.378832	8.816289	-7.950386
F	15.404004	9.207504	-8.755676
H	17.278672	12.323913	-3.806141
H	16.567692	9.94965	2.95678
H	10.123021	9.008743	1.201982
H	12.99695	7.2789	-5.144699
Cl	16.818868	12.776868	-1.082291
Cl	17.834528	10.254543	0.494289
Cl	10.229049	9.25842	-1.587874
Cl	11.856553	6.776198	-2.653904

Final Single Point Energy -3575.77676843521E_h

Coordinates of Compound 5b (C_2F_5-TAPP-Cl_4)

Atomic Type	Coordinates [Å] X	Y	Z
F	11.030829	9.984415	5.686548
F	13.129135	9.821167	6.304988
N	11.662435	9.195141	3.133142
N	13.89638	9.715321	3.80474
C	12.021131	9.247384	1.841344
C	11.046679	9.078395	0.805351
C	11.43825	9.056276	-0.505927
C	12.83594	9.142476	-0.899614
C	13.758935	9.592682	0.101584
C	13.355911	9.556499	1.466059
C	14.275649	9.804664	2.516875
C	15.640528	10.062994	2.176739
C	16.022769	10.162302	0.863934
C	13.375333	8.845315	-2.182752
C	12.622078	9.419424	4.028668
C	12.185414	9.306999	5.493563
N	16.477503	10.219751	-5.669335
N	14.813059	8.565598	-6.126668
C	16.081217	10.282682	-4.386194
C	16.671768	11.225114	-3.484027
C	16.293252	11.245924	-2.166933
C	15.342048	10.294577	-1.614904

C	14.570687	9.54239	-2.561114
C	15.0047	9.485268	-3.91511
C	14.3789	8.628463	-4.85849
C	13.326564	7.768187	-4.409546
C	12.866554	7.860057	-3.123757
C	15.075219	10.054499	-0.233457
C	15.829042	9.360912	-6.448631
C	16.277065	9.28166	-7.911076
F	17.552207	9.69687	-8.042862
F	16.195382	8.006767	-8.34806
H	17.370407	11.962018	-3.881687
H	16.374849	10.115697	2.981022
H	9.993665	9.022096	1.083058
H	12.947767	7.0064	-5.091651
Cl	16.806122	12.608056	-1.211899
Cl	17.73578	10.188579	0.543742
Cl	10.191746	9.14802	-1.720127
Cl	11.801711	6.596426	-2.575294
C	15.403035	10.152003	-8.854824
F	15.483995	11.436069	-8.490016
F	15.818521	10.035548	-10.114956
F	14.124431	9.774573	-8.792667
C	11.952871	7.841344	5.951135
F	10.985214	7.261689	5.241895
F	13.079427	7.136706	5.785167
F	11.610645	7.811771	7.239203

Final Single Point Energy	-4051.28055243311 E_h	
HOMO:	-0.252173 E_h	-6.8620 eV
LUMO:	-0.152585 E_h	-4.1520 eV

Coordinates of Compound 5b⁻ (C_2F_5-TAPP-Cl_4)

Atomic Type	Coordinates [Å] X	Y	Z
F	10.866074	10.455061	5.334804
F	12.910057	10.565927	6.0978
N	11.639126	9.157245	3.104613
N	13.87201	9.694759	3.796664
C	12.054543	9.004859	1.819423
C	11.142071	8.691203	0.783073
C	11.584096	8.556018	-0.524864
C	12.955777	8.692177	-0.907867
C	13.84489	9.186778	0.107999

C	13.41239	9.244834	1.464285
C	14.315555	9.566779	2.519049
C	15.688316	9.712419	2.196815
C	16.084801	9.742918	0.870083
C	13.527613	8.45551	-2.222619
C	12.570662	9.516863	3.979425
C	12.048856	9.793085	5.39634
N	16.34541	10.379216	-5.718843
N	14.787974	8.636953	-6.261911
C	16.017068	10.287683	-4.404069
C	16.596033	11.152788	-3.442959
C	16.283214	11.006081	-2.101175
C	15.411874	9.986151	-1.60756
C	14.656422	9.264959	-2.593348
C	15.030909	9.35648	-3.965244
C	14.426626	8.529453	-4.956237
C	13.499548	7.546007	-4.525553
C	13.100143	7.502479	-3.199085
C	15.162937	9.65549	-0.21834
C	15.713809	9.546973	-6.536912
C	16.115869	9.665969	-8.013419
F	17.427936	9.98557	-8.126322
F	15.911909	8.497373	-8.667389
H	17.254977	11.952765	-3.779707
H	16.416734	9.799536	3.002845
H	10.082487	8.605104	1.022583
H	13.140552	6.806662	-5.241445
Cl	16.869649	12.271256	-1.037266
Cl	17.814292	9.731229	0.568642
Cl	10.3197	8.399176	-1.732247
Cl	12.185738	6.085083	-2.714991
C	15.335308	10.74327	-8.808457
F	15.569812	11.966588	-8.327556
F	15.705566	10.731618	-10.097437
F	14.021404	10.50536	-8.753282
C	11.80627	8.52904	6.258023
F	10.882172	7.73606	5.71481
F	12.938049	7.82756	6.394544
F	11.385523	8.8808	7.481461

Final Single Point Energy -4051.39883207785 E_h

Coordinates of Compound 5c (C_3F_7-TAPP-Cl_4)

Atomic Type	Coordinates [Å]		
	X	Y	Z
F	11.838829	10.242876	5.781019
N	11.937649	8.864832	3.14129
N	14.170651	9.392075	3.796202
C	12.28791	8.921428	1.848353
C	11.300677	8.771459	0.82212
C	11.673456	8.771993	-0.494466
C	13.065503	8.861915	-0.906471
C	14.008145	9.273538	0.093608
C	13.622296	9.220964	1.462737
C	14.551702	9.463391	2.506289
C	15.919353	9.692584	2.156345
C	16.288824	9.787642	0.83886
C	13.575098	8.622292	-2.212825
C	12.899959	9.092351	4.030784
C	12.437317	9.060091	5.487912
N	16.462605	10.310272	-5.737336
N	14.821318	8.645055	-6.22581
C	16.156381	10.244498	-4.428588
C	16.804463	11.101723	-3.483086
C	16.501314	11.013038	-2.148218
C	15.563386	10.03362	-1.623528
C	14.756384	9.344377	-2.588289
C	15.121724	9.39205	-3.963088
C	14.458448	8.597853	-4.935602
C	13.444345	7.685589	-4.498162
C	13.038431	7.688946	-3.191291
C	15.324474	9.728813	-0.24816
C	15.782249	9.507878	-6.547356
F	11.5098	8.089093	5.628893
C	16.197488	9.509884	-8.026105
F	17.171213	10.417255	-8.237223
F	16.684208	8.279481	-8.324668
H	17.488889	11.864837	-3.85493
H	16.663102	9.721106	2.953334
H	10.251358	8.717817	1.113745
H	13.0454	6.964291	-5.211875
Cl	17.098625	12.281881	-1.11674
Cl	17.995005	9.749284	0.489051
Cl	10.40746	8.895288	-1.684798
Cl	12.012477	6.381118	-2.673032
C	13.509942	8.771828	6.591325
F	12.820293	8.657008	7.745695
F	14.09012	7.583331	6.327191

C	15.07672	9.794371	-9.076633
F	15.598525	9.588952	-10.30348
F	14.05722	8.936986	-8.905962
C	14.646907	9.829416	6.842353
F	14.249484	11.056376	6.502101
F	14.953125	9.832375	8.14307
F	15.75438	9.531264	6.170338
C	14.502561	11.23879	-9.059352
F	14.130343	11.577939	-7.815367
F	15.406269	12.11576	-9.489762
F	13.430496	11.302983	-9.846612

Final Single Point Energy -4526.88967085511 E_h
HOMO: -0.253877 E_h -6.9084 eV
LUMO: -0.154487 E_h -4.2038 eV

Coordinates of Compound 5c⁻ (C_3F_7-TAPP-Cl_4)

Atomic Type	Coordinates [Å]		
	X	Y	Z
F	11.542008	9.849499	5.758376
N	11.95362	8.817532	3.171292
N	14.227504	9.169702	3.841412
C	12.312162	8.864737	1.863864
C	11.341469	8.780272	0.835724
C	11.736835	8.755245	-0.491091
C	13.10748	8.774967	-0.895556
C	14.063152	9.122613	0.119219
C	13.672701	9.070813	1.4892
C	14.623114	9.22803	2.538309
C	15.985243	9.378825	2.181434
C	16.34786	9.500048	0.84712
C	13.623445	8.531986	-2.227274
C	12.933829	8.967763	4.051575
C	12.466192	8.885943	5.50364
N	16.467448	10.337031	-5.750261
N	14.812119	8.684097	-6.275455
C	16.184456	10.219418	-4.425329
C	16.849983	11.013452	-3.45946
C	16.570447	10.851773	-2.110323

C	15.640847	9.885283	-1.61175
C	14.812673	9.246904	-2.59839
C	15.157195	9.340852	-3.977289
C	14.475406	8.573717	-4.966265
C	13.48109	7.658516	-4.537561
C	13.087021	7.643418	-3.210434
C	15.400363	9.524603	-0.222891
C	15.76253	9.565399	-6.564519
F	11.855207	7.687651	5.711843
C	16.140606	9.635124	-8.050577
F	17.099201	10.565346	-8.26496
F	16.643724	8.430256	-8.431954
H	17.551157	11.77668	-3.796334
H	16.743125	9.364372	2.96466
H	10.285841	8.765336	1.106448
H	13.048112	6.965876	-5.258983
Cl	17.281163	12.047092	-1.041262
Cl	18.06906	9.439548	0.509448
Cl	10.453414	8.886453	-1.68123
Cl	11.9998	6.349425	-2.741814
C	13.539107	8.981468	6.633438
F	12.85147	8.886339	7.798729
F	14.366316	7.923571	6.565316
C	14.999038	9.949788	-9.069353
F	15.499923	9.788811	-10.319468
F	13.975683	9.093333	-8.927757
C	14.402109	10.296015	6.724365
F	13.856007	11.294945	6.027387
F	14.470762	10.690913	8.005351
F	15.645777	10.095346	6.304116
C	14.424988	11.39294	-9.012874
F	14.050635	11.717851	-7.772698
F	15.323443	12.282867	-9.439016
F	13.350441	11.472074	-9.806339

Final Single Point Energy -4527.01117312327 E_h

Coordinates of Compound 5d (H-TAPP-Cl_4)

Atomic Type	Coordinates [Å]		
	X	Y	Z
N	11.839425	9.102857	3.210117
N	14.082235	9.651397	3.845876
C	12.177842	9.179289	1.912358
C	11.186042	9.019376	0.890981

C	11.543419	9.04734	-0.430053
C	12.925859	9.181145	-0.85903
C	13.867608	9.593683	0.141385
C	13.499645	9.510477	1.514481
C	14.436916	9.751287	2.55295
C	15.797209	10.013186	2.186638
C	16.146077	10.146226	0.869398
C	13.429483	8.981177	-2.177116
C	12.810503	9.334376	4.095546
N	16.347157	10.724039	-5.663865
N	14.70876	9.058904	-6.193731
C	16.007404	10.660312	-4.365421
C	16.623573	11.530947	-3.408329
C	16.306265	11.429339	-2.080266
C	15.388396	10.423088	-1.572832
C	14.593938	9.732796	-2.546885
C	14.971173	9.798028	-3.918445
C	14.328058	9.006014	-4.905652
C	13.318699	8.081168	-4.479479
C	12.910699	8.057238	-3.172633
C	15.16888	10.086538	-0.205141
C	15.686715	9.915649	-6.494827
H	17.294904	12.310084	-3.770551
H	16.551739	10.03762	2.973653
H	10.141208	8.935262	1.191523
H	12.931401	7.362139	-5.201995
Cl	16.855879	12.707472	-1.029034
Cl	17.850633	10.154263	0.493314
Cl	10.254514	9.155001	-1.601849
Cl	11.910924	6.715409	-2.678602
H	15.988571	9.958973	-7.549045
H	12.528546	9.253682	5.153174

Final Single Point Energy	- 2901.49261065712 E_h	
HOMO:	-0.236111 E_h	-6.4249 eV
LUMO:	-0.136157 E_h	-3.7050 eV

Coordinates of Compound 5d⁻ (H-TAPP-Cl_4)

Atomic Type	Coordinates [Å] X	Y	Z
N	11.811966	9.159417	3.236398
N	14.080059	9.631735	3.877327
C	12.15499	9.227465	1.924782
C	11.180784	9.086064	0.902874
C	11.563619	9.079163	-0.427803

C	12.925019	9.182373	-0.849488
C	13.866744	9.584624	0.158733
C	13.494954	9.510754	1.532389
C	14.447018	9.715734	2.572299
C	15.797885	9.940498	2.198849
C	16.135691	10.089608	0.864272
C	13.448712	8.95971	-2.183795
C	12.797061	9.359849	4.110404
N	16.321498	10.768306	-5.698452
N	14.723278	9.050392	-6.222978
C	15.984603	10.699618	-4.385236
C	16.576737	11.565906	-3.429229
C	16.268976	11.430387	-2.085944
C	15.388284	10.424279	-1.582103
C	14.603052	9.726764	-2.562405
C	14.974559	9.79969	-3.936129
C	14.34926	8.979321	-4.91939
C	13.375309	8.042673	-4.481945
C	12.96466	8.034171	-3.159506
C	15.175331	10.061105	-0.193546
C	15.673215	9.938337	-6.513966
H	17.243723	12.355254	-3.775749
H	16.56283	9.959255	2.97539
H	10.13073	9.009173	1.18542
H	12.983856	7.315307	-5.193254
Cl	16.873692	12.696331	-1.026492
Cl	17.857539	10.129804	0.503709
Cl	10.255801	9.123529	-1.604529
Cl	11.939489	6.687034	-2.682432
H	15.969102	9.991744	-7.572954
H	12.510269	9.290408	5.171091
N	11.811966	9.159417	3.236398
N	14.080059	9.631735	3.877327
C	12.15499	9.227465	1.924782
C	11.180784	9.086064	0.902874
C	11.563619	9.079163	-0.427803
C	12.925019	9.182373	-0.849488

Final Single Point Energy -2901.59306515177 E_h

TFT Fabrication Process

The TFTs were fabricated in the bottom-gate, top-contact (inverted staggered) device architecture on heavily doped silicon substrates that also serve as a global gate electrode for all TFTs on the substrate. The gate dielectric is a stack of silicon dioxide (SiO_2) with a thickness of 100 nm grown by thermal oxidation in dry oxygen and aluminum oxide (Al_2O_3) with a thickness of 8 nm deposited by atomic layer deposition (Savannah 100, Cambridge NanoTech Inc.) at a substrate temperature of 250 °C. The Al_2O_3 surface was activated by oxygen plasma (Oxford Instruments) and then passivated with a self-assembled monolayer (SAM) by immersing the substrate into a 1-mM solution of n-tetradecylphosphonic acid (PCI Synthesis, Newburyport, MA, U.S.A.) in 2-propanol (VLSI grade) for one to two hours. The substrates were then rinsed in 2-propanol and dried on a hotplate at a temperature of 150 °C for 1 min. The resulting SiO_2/Al_2O_3/SAM gate dielectric has a total thickness of 110 nm and a unit-area capacitance of 34 nF/cm^2.[S1] The organic semiconductors were deposited onto the gate dielectric by sublimation in vacuum (Leybold UNIVEX 300) with a background pressure of about 10^{-6} mbar, at a substrate temperature of 70 °C, with a deposition rate of about 1 nm/min and with a nominal thickness of 30 nm. Finally, gold source and drain contacts were deposited onto the organic semiconductor layer by thermal evaporation in vacuum (Leybold UNIVEX 300) and patterned using a polyimide shadow mask. The TFTs have a channel length of 100 µm and a channel width of 200 µm. The current-voltage characteristics of the TFTs were measured in ambient air at room temperature.

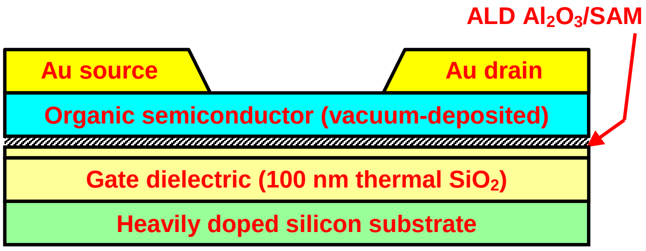

Crystal Structures of Compounds 5a-d

X-ray Crystal Structure Determinations

Crystal data and details of the structure determinations are compiled in Table S1. Full shells of intensity data were collected at low temperature with a Bruker AXS Smart 1000 CCD diffractometer (Mo-K_α radiation, sealed X-ray tube, graphite monochromator; compound **5b**) or an Agilent Technologies Supernova-E CCD diffractometer (Cu-K_α radiation, microfocus X-ray tube, multilayer mirror optics; compounds **5a** and **5c**·$CHCl_3$). Detector frames (typically ω-, occasionally φ-scans, scan width 0.4...1°) were integrated by profile fitting.[S2-S4] Data were corrected for air and detector absorption, Lorentz and polarization effects[S3,S4] and scaled essentially by application of appropriate spherical harmonic functions.[S5-S7] Absorption by the crystal was treated with a semiempirical multiscan method (as part of the scaling process), and augmented by a spherical correction,[S5-S7] or numerically (Gaussian grid).[S6,S7] For datasets collected with the microfocus tube an illumination correction was performed as part of the numerical absorption correction.[S8] The structures were

solved by the heavy atom method combined with structure expansion by direct methods applied to difference structure factors (compound **5a**)[S9] or by the charge flip procedure (compounds **5b** and **5c**·$CHCl_3$)[S10] and refined by full-matrix least squares methods based on F^2 against all unique reflections.[S11] All non-hydrogen atoms were given anisotropic displacement parameters. Hydrogen atoms on the peropyrene core were fully refined with isotropic displacement parameters. The chloroform hydrogen in **5c**·$CHCl_3$ was refined riding.[S12]

CCDC 1908780 - 1908782 contains the supplementary crystallographic data for this paper. These data can be obtained free of charge from the Cambridge Crystallographic Data Centre's and FIZ Karlsruhe’s joint Access Service via https://www.ccdc.cam.ac.uk/structures/?.

Crystals of compounds **5a** and **5c** were grown by slow evaporation of saturated solutions of the compounds in $CHCl_3$. Crystals of **5b** were be grown by slow evaporation of a toluene solution.

Compound 5a (CF_3-TAPP-Cl_4)

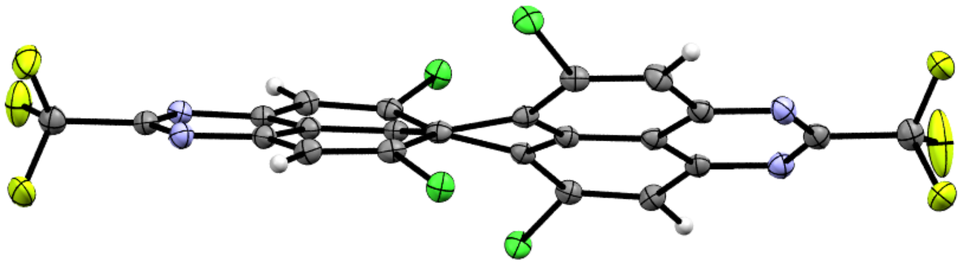

Ortep view (50% probability level) of 2 (C = grey ,N = blue, F = yellow, Cl = green, H = white).

Compound 5b (C_2F_5-TAPP-Cl_4)

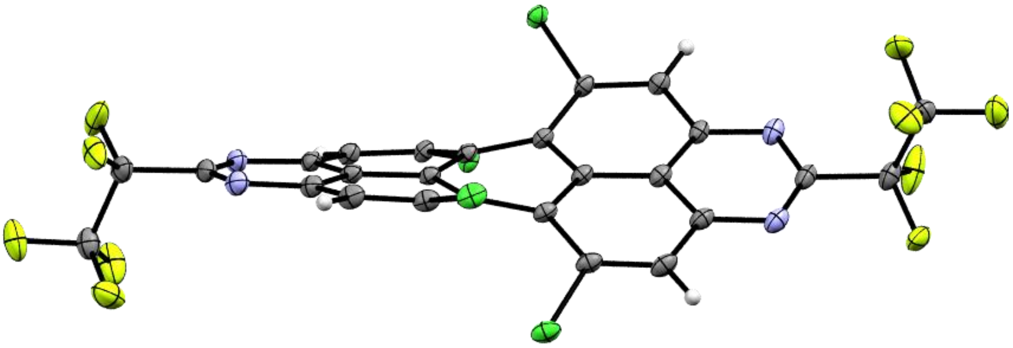

Ortep view (50% probability level) of 2 (C = grey ,N = blue, F = yellow, Cl = green, H = white).

Compound 5c (C_3F_7-TAPP-Cl_4)

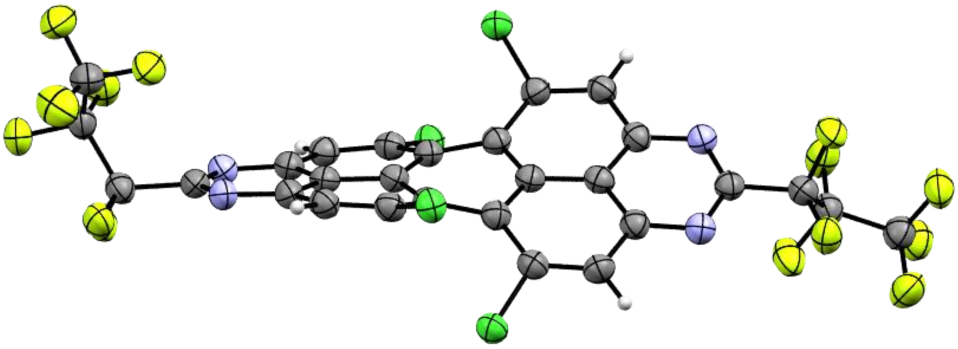

Ortep view (50% probability level) of 2 (C = grey ,N = blue, F = yellow, Cl = green, H = white).

Table S1. Details of the crystal structure determinations of compounds **5a**, **5b** and **5c**·$CHCl_3$.

	**5a**	**5b**	**5c**· $CHCl_3$
formula	$C_{24}H_4Cl_4F_6N_4$	$C_{26}H_4Cl_4F_{10}N_4$	$C_{29}H_5Cl_7F_{14}N_4$
crystal system	orthorhombic	monoclinic	triclinic
space group	*P bca*	*I* 2/*a*	*P* -1
a /Å	15.09084(13)	12.532(6) [b]	9.8735(4)
b /Å	12.56233(15)	13.358(6) [b]	12.5112(3)
c /Å	22.1683(2)	15.002(6) [b]	13.9911(6)
α /°			67.809(3)
β /°		98.345(11) [b]	83.349(4)
γ /°			78.762(3)
V /$Å^3$	4202.59(7)	2484.7(19) [b]	1567.91(11)
Z	8	4	2
M_r	604.11	704.13	923.52
F_{000}	2384	1384	904
d_c /Mgm^{-3}	1.910	1.882	1.956
μ /mm^{-1}	5.863	0.581	6.882
max., min. transmission factors	1.000, 0.598 [a]	0.7464, 0.6615 [c]	1.000, 0.8232 [c]
X-radiation, λ /Å	Cu-K_α, 1.54184	Mo-$K\alpha$, 0.71073	Cu-K_α, 1.54184
data collect. temperat. /K	120(1)	100(1)	120(1)
θ range /°	4.0 to 70.5	2.1 to 32.5	3.4 to 71.2
index ranges *h*,*k*,*l*	±18, ±15, ±26	±18, ±19, -21 ... 22	±12, ±15, -17 ... 16
reflections measured	130039	31427	52291
unique [R_{int}]	4000 [0.0649]	4283 [0.0298]	6004 [0.0500]
observed ($I \geq 2\sigma(I)$)	3508	3813	4793
data / restraints /parameters	4000 / 0 / 359	4283 / 0 / 207	6004 / 0 / 503
GooF on F^2	1.062	1.046	1.033
R indices ($F>4\sigma(F)$) $R(F)$, $wR(F^2)$	0.0305, 0.0748	0.0334, 0.0914	0.0504, 0.1338
R indices (all data) $R(F)$, $wR(F^2)$	0.0370, 0.0776	0.0384, 0.0965	0.0650, 0.1447
largest residual peaks /$eÅ^{-3}$	0.651, -0.250	0.677, -0.305	0.568, -0.434
CCDC deposition number	1908780	1908781	1908782

[a] numerical absorption correction; [b] includes systematic error contributions from Monte Carlo simulations; [c]

References

S1 R. Hofmockel, U. Zschieschang, U. Kraft, R. Rödel, N. H. Hansen, M. Stolte, F. Würthner, K. Takimiya, K. Kern, J. Pflaum, H. Klauk, *Org. Electron.*, **2013**, *14*, 3213-3221

S2 K. Kabsch, in: M. G. Rossmann, E. Arnold (eds.), *"International Tables for Crystallography" Vol. F*, Ch. 11.3, Kluwer Academic Publishers, Dordrecht, The Netherlands, **2001**.

S3 *SAINT*, Bruker AXS GmbH, Karlsruhe, Germany **1997-2017**.

S4 *CrysAlisPro*, Agilent Technologies UK Ltd., Oxford, UK **2011-2014** and Rigaku Oxford Diffraction, Rigaku Polska Sp.z o.o., Wrocław, Poland **2015-2019**.

S5 R. H. Blessing, *Acta Cryst.* **1995**, *A51*, 33.

S6 (a) G. M. Sheldrick, *SADABS*, Bruker AXS GmbH, Karlsruhe, Germany **2004-2014**; (b) L. Krause, R. Herbst-Irmer, G. M. Sheldrick, D. Stalke, *J. Appl. Cryst.* **2015**, *48*, 3.

S7 *SCALE3 ABSPACK*, *CrysAlisPro*, Agilent Technologies UK Ltd., Oxford, UK **2011-2014** and Rigaku Oxford Diffraction, Rigaku Polska Sp.z o.o., Wrocław, Poland **2015-2019**.

S8 W. R. Busing, H. A. Levy, *Acta Cryst.* **1957**, *10*, 180.

S9 (a) P. T. Beurskens, G. Beurskens, R. de Gelder, J. M. M. Smits, S. Garcia-Granda, R. O. Gould, *DIRDIF-2008*, Radboud University Nijmegen, The Netherlands, **2008**; (b) P. T. Beurskens, in: G. M. Sheldrick, C. Krüger, R. Goddard (eds.), *Crystallographic Computing 3*, Clarendon Press, Oxford, UK, **1985**, p. 216.

S10 (a) L. Palatinus, *SUPERFLIP*, EPF Lausanne, Switzerland and Fyzikální ústav AV ČR, v. v. i., Prague, Czech Republic, **2007-2014**; (b) L. Palatinus, G. Chapuis, *J. Appl. Cryst.* **2007**, 40, 786.

S11 (a) G. M. Sheldrick, *SHELXL-20xx*, University of Göttingen and Bruker AXS GmbH, Karlsruhe, Germany **2012-2018**; (b) W. Robinson, G. M. Sheldrick in: N. W. Isaaks, M. R. Taylor (eds.) „*Crystallographic Computing 4*“, Ch. 22, IUCr and Oxford University Press, Oxford, UK, **1988**; (c) G. M. Sheldrick, *Acta Cryst.* **2008**, *A64*, 112; (d) G. M. Sheldrick, *Acta Cryst.* **2015**, *C71*, 3.

S12 (a) J. S. Rollett in: F. R. Ahmed, S. R. Hall, C. P. Huber (eds.) „*Crystallographic Computing*“ p. 167, Munksgaard, Copenhagen, Denmark, **1970**; (b) D. Watkin in: N. W. Isaaks, M. R. Taylor (eds.) „*Crystallographic Computing 4*“, Ch. 8, IUCr and Oxford University Press, Oxford, UK, **1988**; (c) P. Müller, R. Herbst-Irmer, A. L. Spek, T. R. Schneider, M. R. Sawaya in: P. Müller (ed.) *“Crystal Structure Refinement”*, Ch. 5, Oxford University Press, Oxford, UK, **2006**; (d) D. Watkin, *J. Appl. Cryst.* **2008**, *41*, 491.
